# Formalizing the fundamental Faustian bargain: Inefficacious decision-makers sacrifice their freedom of choice to coercive leaders for economic security

**DOI:** 10.1371/journal.pone.0275265

**Published:** 2022-09-27

**Authors:** Daniel A. DeCaro, Marci S. DeCaro, Jared M. Hotaling, Rachel Appel

**Affiliations:** 1 Department of Psychological and Brain Sciences, University of Louisville, Louisville, KY, United States of America; 2 Department of Urban and Public Affairs, University of Louisville, Louisville, KY, United States of America; 3 Department of Psychology, University of Illinois at Urbana-Champaign, Urbana-Champaign, IL, United States of America; Federal University of Paraiba, BRAZIL

## Abstract

Individuals typically prefer the freedom to make their own decisions. Yet, people often trade their own decision control (procedural utility) to gain economic security (outcome utility). Decision science has not reconciled these observations. We examined how decision-makers’ efficacy and security perceptions influence when, why, and how individuals exchange procedural and outcome utility. Undergraduate adults (*N* = 77; *M*_age_ = 19.45 years; 73% female; 62% Caucasian, 13% African American) were recruited from the psychology participant pool at a midwestern U.S. metropolitan university. Participants made financial decisions in easy and hard versions of a paid card task resembling a standard gambling task, with a learning component. During half the trials, they made decisions with a No-Choice Manager who controlled their decisions, versus a Choice Manager who granted decision control. The hard task was designed to be too difficult for most participants, undermining their efficacy and security, and ensuring financial losses. The No-Choice Manager was designed to perform moderately well, ensuring financial gains. Participants felt greater outcome satisfaction (utility) for financial gains earned via Choice, but not losses. Participants (85%) preferred the Choice manager in the easy task but preferred the No-Choice Manager (56%) in the hard task. This change in preference for choice corresponded with self-efficacy and was mediated by perceived security. We used Decision Field Theory to develop potential cognitive models of these decisions. Preferences were best described by a model that assumed decision-makers initially prefer Choice, but update their preference based on loss-dependent attentional focus. When they earned losses (hard task), decision-makers focused more on economic payoffs (financial security), causing them to deemphasize procedural utility. Losses competed for attention, pulling attention toward economic survivability and away from the inherent value of choice. Decision-makers are more likely to sacrifice freedom of choice to leaders they perceive as efficacious to alleviate perceived threats to economic security.

## Introduction

Decades of research demonstrates that self-determination—whether individual or collective [1,2]—is a fundamental human need [3,4]. The ability to exercise choice is associated with cognitive and psychosocial benefits, including positive affect, intrinsic motivation, satisfaction, cooperation, and mental health [5,6]. As such, decision control is typically highly valued, especially in Western cultures (e.g., United States), which commonly equate freedom with individual choice [e.g., 7–10].

This observation is sometimes treated as a core principle of human nature [5,8]. However, these findings have not been sufficiently reconciled with equal evidence demonstrating undesirability of choice [e.g., 7,11, cf. 12–15]. This observation also contrasts with lay experience, and a foundational principle in political science: societies exist because individuals sacrifice some freedom of choice to others (e.g., leaders, mentors, families, employers, governments) in exchange for security, economic welfare, and goods and services that benefit survival [16,17, cf. 18].

This tradeoff between decision control and economic security has been described as a *Faustian bargain* [18,19]. Although inherent to human decision-making and integral to human civilization, the decision calculus behind this tradeoff has been largely ignored in decision science [8,13]. Thus, there is substantial evidence indicating that the tradeoff exists, but much less formalization of the decision-processes involved. This scientific gap is important. The bargain must be understood to account for the nature and rise of human institutions (e.g., employment, government), policy preferences, and motivation and behavior within rule-governed systems (e.g., compliance, cooperation) [8,20–22].

Early research demonstrates a tendency for people who value choice to abandon it when faced with dire, tragic, or treacherous prospects (i.e., losses), especially when they feel inefficacious to mitigate negative outcomes themselves [e.g., 11,13, cf. 12,15]. Individuals may fail to act, leave the decision to chance or fate, engage in self-enhancing rationalizations, or relinquish decision control to more efficacious others (benevolent dictators) for security [12,15,23]. In egregious situations, individuals may relinquish control to coercive dictators, who offer security at the cost of fundamental liberties [24–29].

Decision science based on economic rational choice theory [30,31] assumes that such decisions are based exclusively on the anticipated economic outcomes [8]. Rational decision-makers should simply focus on the instrumental economic outcomes, ignoring preference for choice. Recent research on the concept of *procedural utility* (utility derived from exercising choice), suggests a more nuanced approach [8]. When freedom of choice is costly or risky, some individuals still prefer personal decision control (e.g., self-employment), whereas others do not (e.g., traditional employment). DeCaro et al. (2020) [13] demonstrated that antipathy for losses plays an important role, with individuals generally preferring choice when earning gains, but preferring to relinquish control to others if doing so prevents losses. The motivational and cognitive processes underlying these decisions remains poorly understood.

We build on these concepts to lay foundations for a descriptive, cognitive and utility-driven examination of the tradeoff between decision control and economic security. In the current study, participants chose whether to relinquish decision control to a coercive, but efficacious, No-Choice manager. They made these decisions in easy versus hard decision tasks, designed to decrease self-efficacy, threaten economic security, and ensure financial losses. To gain insight into participants’ motivations, we assessed their self-efficacy and security. We hypothesized that participants would relinquish control to the coercive No-Choice manager in the hard task, when they feel inefficacious and insecure making decisions themselves.

To describe the cognitive and utility processes involved in participants’ preferences for choice, we used Decision Field Theory’s computational cognition framework [32] to create and test diagnostic models of attention-based, utility updating. Specifically, based on prior research suggesting that losses capture attention [33,34], we hypothesized that economic losses compel individuals to focus on economic security (*outcome utility*) instead of personal freedom (*procedural utility*). This shift in attention determines how much procedural utility, versus economic outcome utility, influences preference for choice [cf. 13]. We find support for these hypotheses in the current study. This study adds to the conceptual and methodological rigor of prior research into desire for control [3,12], self-determination [5,35], procedural utility [8], and preference for choice in situations with economic costs for personal choice.

### Trading freedom of choice for security

Exchanges between freedom of choice (or “decision control”) and security are widespread. This tradeoff is thought to be most visible during crisis, for example, when great societal instability (e.g., economic depression) or unsafety (e.g., terrorism), seem to make individuals especially willing to sacrifice liberty for security [27,36–40]. However, the Faustian bargain occurs in any situation where others are more efficacious and possess power/authority [8,12,23], including government [18,19,41], family (parent/child) and education (teacher/student) [35,42], healthcare (doctor/patient) [43], and employment [44].

### Preference for choice

Numerous experiments have examined preference for choice and decision control [3,12]. Generally speaking, individuals prefer control when they feel efficacious and when deciding among positive, rather than negative, alternatives [12,15,45,46]. For example, in a reaction-time task involving the prevention of painful shocks, Miller (1980) [47] found that individuals preferred control when they felt efficacious (fast enough) to prevent the shocks themselves, but otherwise relinquished control to a more efficacious (faster) partner [cf. 48].

Some decision scientists have begun to conceptualize these preferences in terms of tradeoffs between *procedural utility* (i.e., the value individuals place on freedom of choice and fair institutional decision processes), and *outcome utility* (i.e., the value placed on economic outcomes) [8,13]. Most early research in this domain demonstrated a robust, positive value and preference for choice [e.g., 9,44,cf. 8,3]. For example, in a survey of 23 Western countries, Benz and Frey (2008) [44] found that self-employed individuals were more satisfied than traditionally-employed individuals, controlling for workload and earnings. Szrek and Baron (2007) [49] found that individuals preferred, and were willing to pay more for, identical insurance plans presented as choice options instead of policies assigned by their employer. Leotti and Delgado (2014) [10] found similar results in a task involving financial payoffs. These finding suggest a potential boost to utility associated with exercising choice, a procedural utility [50].

### Tradeoffs involving losses and coercive authorities

Many experiments have examined desire for control. However, few experiments have specifically examined procedural utility (i.e., attempting to determine the utility processes and calculus underlying preference for choice) [13]. Most procedural utility experiments examine tradeoffs involving positive financial outcomes, poorly defined outcomes, and/or benevolent dictators—not well-defined losses, or coercive dictators. For example, Leotti and Delgado (2014) [10] attempted to assess the effect of financial losses on preference for choice. However, their implementation lacked financial goals [cf. 51] or any other reference point [cf. 52] to parse outcomes into net losses, versus gains. Additionally, their task did not involve an authority figure. “Choice” was operationalized as choosing from among one option versus a series of options. There was no true Faustian bargain. Tyler (2006) [53] posed two retirement plans to retirees, a high procedural utility plan, with inclusive consultation from a caring advisor and “average” financial return, versus a low procedural utility plan with no special consultation and an “above average” financial return. Most participants (62%) preferred the high procedural utility plan, even though it paid less than the low procedural utility plan. However, the financial prospects did not involve losses, because both plans were at least “above average,” and both advisors were benevolent, not coercive.

These factors—lack of well-defined financial losses and absence of a coercive dictator—overlook critical elements of many important choice tradeoffs in society [12,14,27,38,39], and likely artificially inflate the apparent utility and preference for choice. DeCaro et al. (2020) [13] addressed these concerns by examining coercive exchanges involving well-defined financial losses. *Losses* do not simply refer to negative outcomes: they refer more specifically to outcomes that fall below (i.e., fail to reach) a critical goal/reference point [51,52,54]. Losses capture attention [34], often eliciting a strong, negative affective response [55,56]. Losses signal failure and may therefore be evolutionarily linked to survival; organisms that learn to avoid losses increase their chances for survival [33,54]. This deep-seated antipathy for losses has been described as loss aversion or negativity bias [33,57].

DeCaro et al. (2020) [13] had participants complete a card-based decision task, followed by a choice preference task. The first task was designed to elicit individuals’ outcome satisfaction for financial payoffs designated as losses or gains, to assess felt utility. Participants saw four decks of cards. During each trial, participants drew a card from a deck, with the goal of learning the average payoff of each deck to earn more than the typical (status quo) payment of $5.00. Each card was worth a monetary value, from $1 to $9, representing losses (below $5.00) and gains (above $5.00). After each trial, participants rated their satisfaction with the financial outcome, a measure of felt utility [cf. 50,58]. The task was designed to be moderately difficult, so that most participants could quickly learn the best deck, Deck D. Participants were supervised by automated managers. During half of the trails, a Choice Manager affirmed participants’ right to choose and granted them full decision control (e.g., “I appreciate your perspective on this. Please choose for yourself”). During the other trials, a coercive No-Choice Manager took control (e.g., “When I’m in charge, decisions must be made through me. Choose Deck D.”).

After the card task, participants completed a choice preference task, indicating which manager they would prefer to work for if they did the experiment again. Participants saw a series of “job offers,” with final payoffs. The Choice Manager always offered less than the No-Choice Manager. Thus, if individuals wanted decision control, they had to sacrifice earnings. To gauge the potential influence of losses versus gains, the offers spanned losses (e.g., *Choice $1 vs*. *No-Choice $2*) and gains (e.g., *Choice $8 vs*. *No-Choice $9*).

During the outcome satisfaction task (card task), participants were more satisfied by financial payoffs earned with Choice compared to No-Choice, confirming that procedural utility (derived from freedom of choice) was, indeed, valued beyond outcome utility. However, this effect was greatly reduced for losses (η_p_^2^ = .04), compared to gains (η_p_^2^ = .26). A similar pattern emerged during the choice preference task. When the job offers differed by only $1 (e.g., *Choice $8 vs*. *No-Choice $9*), most participants (64%) preferred choice. However, when the pay difference increased, yielding greater losses (e.g., *Choice $1 vs*. *No-Choice $9*), most participants (e.g., 93%) preferred no-choice. Thus, individuals were more likely to select the No-Choice Manager when doing so provided a way to escape significant financial loss.

DeCaro et al. (2020) hypothesized that decision-makers placed more weight (“attention”) on financial outcomes when facing loss prospects [cf. 34], because losses drew attention away from the procedural utility (freedom of choice) dimension, causing them to prioritize economic welfare over self-determination. However, the authors did not use computational cognitive models to represent and test these assumed processes. DeCaro et al. also did not examine self-efficacy or security perceptions as a potential mechanism of these effects. It is important to consider the involvement of self-efficacy and security because prior theory suggests there is a crucial link [12,25,29,33]. Preference for coercive, authoritarian leaders, norms, and government systems is thought to be based, in part, on the sense of economic welfare and security those regimes promise to provide to inefficacious individuals [27,28,38,59]. Hence, the prospect of economic security may outweigh the prospect of procedurally fair or autonomy-supportive leaders and decision processes. We address these issues in the current study.

### Current study

We extended DeCaro et al.’s (2020) [13] laboratory experiment, using a similar card-based decision task to assess outcome satisfaction and preference for choice as a function of task difficulty (easy, hard) and corresponding effects to efficacy and security. We predicted that individuals would report greater satisfaction for financial payoffs earned via choice, primarily among gains but not losses, replicating DeCaro et al. (2020). However, we also expected subsequent preference for choice to be moderated by task difficulty. We hypothesized that participants would prefer the Choice Manager on the easy task, which they felt efficacious to perform themselves. In contrast, we expected participants to prefer the coercive, No-Choice Manager on the hard task, which they felt inefficacious to perform. We expected these effects to be mediated by security. Thus, individuals should prefer the No-Choice Manager during the hard task, in part, because this manager provides greater security.

We hypothesized attentional focus to be an important cognitive mechanism underlying preference for choice in the easy versus hard task. To investigate this possibility, we used Decision Field Theory’s [32] attention-based utility framework to test a novel procedural utility model, with a central attention mechanism. Specifically, participants should pay greater attention to the procedural utility dimension in the easier, more secure task, and relatively less attention in the harder, less secure task. In more practical terms, when individuals decide which manager they prefer on the harder task, they should focus more on the utility they derive from good outcomes (outcome utility), not the utility they derive from exercising freedom of choice (procedural utility). In essence, we expected participants’ attention to be consumed by economic considerations, overshadowing considerations for personal freedom or decisional control.

## Materials and methods

Participants completed a modified version of DeCaro et al.’s (2020) [13] card-based decision task, involving an easy and hard version of the task. Participants indicated which manager they would prefer to work with (Choice, No-Choice) on the easy versus hard task. We used participants’ self-reported self-efficacy and security perceptions to test the involvement of these psychological factors. Finally, we used participants’ earnings in the card-based decision task, and their ratings of manager procedural justice and self-determination, as measures of economic and procedural utility, to develop and test a novel cognitive model. Due to their complexity, we describe the major details of this study in the following sections. Additional methodological details, and specific stimuli/instructions can be found in the Supporting Information (see *S1*.*0 Study Materials* in S1 File).

### Participants

Eighty-two individuals were recruited to participate in this study. The final sample consisted of 77 undergraduate students (age: *M* = 19.45 years, *SD* = 2.56; 73% female; 62% Caucasian, 13% African American, 8% Biracial, 7% Asian, 4% Hispanic) recruited from the intro psychology participant pool maintained at a Midwest metropolitan university in the U.S (see *Statistical methods and analyses* for sampling criterion and data exclusions). Individuals participated for partial course credit, as well as any payments earned during the study. Participants were paid their average earnings in the decision task (*M* = $5.35, *95%CI*[$5.30, $5.39]). Any individual at least 18 years old and able to follow verbal and written instructions in English were eligible to participate. This study was conducted for approximately three months, from September to November 2020.

### Design

We tested our hypotheses using a 2 (choice procedure: choice, no-choice) × 2 (outcome valence: losses, gains) × 2 (difficulty: easier, harder) within-subjects design. Order of task difficulty was counterbalanced between-subjects (order: easier/harder *n =* 40, harder/easier *n =* 37).

### Procedure

All materials were presented to participants separately, on an individual computer in a private workspace. Participants completed the card-based decision tasks and preference task after informed consent. Participants completed measures of perceived self-efficacy, security, and need satisfaction (i.e., self-determination, security) after each card-based decision task. Participants completed a final demographics survey, were paid, and debriefed. The study lasted approximately one hour.

### Card task

The purpose of the card task was to give participants experience with each manager in the easy and hard performance environments, and to assess participants’ outcome satisfaction prior to the preference task. If procedural utility, indeed, matters, then participants should report greater satisfaction with financial payoffs earned via choice. If negativity bias or loss aversion also matters, then this satisfaction should be contingent upon valence (i.e., loss, gain).

**Fig 1:**
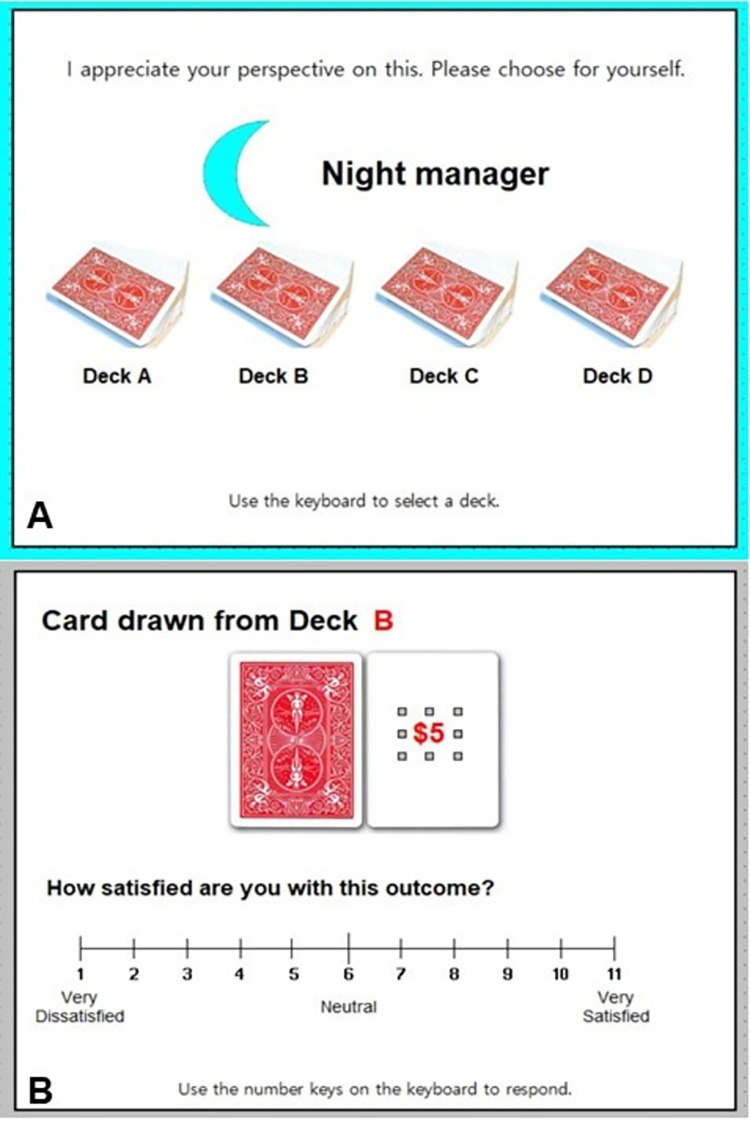
Decision trial in the outcome-satisfaction (card) task. (A) A choice trial is depicted. (B) Participants reported their outcome satisfaction immediately afterward on a subsequent screen.

Participants were informed that they would complete a card-based decision task and be paid between $1 to $9 based on their overall (i.e., average) performance. They were told that they would see four card decks during each trial (Fig 1) and that each card they drew had a payoff from $1 to $9. For the easy task, participants had to discover which deck generated the highest payoffs. For the hard task, they had to discover a sequence, selecting cards from the decks in the proper order to generate the highest payoffs. To establish a status quo economic goal (i.e., reference point), we informed participants that “the majority of people scored an average of $5” on previous (pilot) tests and that scores above/below $5 should be considered indicators of better/worse performance [cf. 13,51]. Thus, scores below $5 were considered losses, and scores above $5 were considered gains, although we did not use these terms with the participants.

After reading the task instructions, participants completed a quiz assessing their understanding of the task, payment, and status quo goal/reference point (i.e., the “average” earnings participants can expect with typical performance). The computer provided feedback on the quiz, including correct answers for questions answered incorrectly. This feedback was given to ensure that participants understood the payoff scale ($1 through $9), status quo ($5), and the fact that their payment would be contingent on their performance (i.e., average earnings).

#### Easy task

During the easy task block, each deck had an undisclosed average payout: *Decks A* and *C* $2 (losses), *Deck B* $5 (status quo), and *Deck D* $8.70 (gains; see *S1*.*2*.*1 Decision Trials and Payoffs*: *Easy Task* in S1 File for details). Participants were instructed to identify which deck(s) yielded the best payoffs. This version of the task replicates the moderate difficulty of DeCaro et al.’s (2020) original task: challenging but learnable, so that most participants would feel efficacious.

#### Hard task

During the hard task block, participants needed to discover a complex, 9-sequence pattern. Specifically, to achieve the highest payoffs they had to draw from the correct deck on the correct trial (e.g., Trial 1: Deck B, Trai1 2: Deck A), or “B, A, C, D, D, A, C, B, A” for short. Sequence-learning research and pilot testing indicated that this sequence would be extremely difficult to learn, given its length and task complexity (cf. Raw et al., 2019).

Correct trials yielded outcomes drawn from status quo and gains pools, $4 through $9 (mimicking Decks B and D in the easy task). Incorrect trails yielded outcomes drawn from the losses pool, $1 through $3 (mimicking Decks A and C in the easy task). The payoffs of these pools were carefully calibrated to exactly match those in the easy task (see *S1*.*2*.*2 Decision Trials and Payoffs*: *Hard Task* in S1 File). Thus, it was possible for individuals to earn the same overall payoffs in both tasks. However, earning the same in both versions of the task was very unlikely because, as described further below, the hard task was designed to be so difficult that most participants should earn losses, on average.

#### Order

The order of the easy and hard tasks was counterbalanced across participants, with half of the participants receiving the easy task first. The tasks were referred to as Parts 1 and 2 during the task instructions and follow-up questions.

#### Choice

As in DeCaro et al.’s (2020) original experiment, each trial was accompanied by “guidance” from automated managers, identified simply as “Day” and “Night” managers (Fig 1). The Choice Manager (Night) let participants decide for themselves. Participants selected a deck (A, B, C, or D) using labeled keys on the computer keyboard. Manager guidance appeared at the top of the screen, randomly selected from a shortened list of statements originally used by DeCaro et al. The statements emphasized participants’ choice (e.g., “Feel free to handle this decision yourself”; see Supporting Information). The No-Choice Manager (Day) told participants to choose a particular deck (e.g., “When I’m in charge, decisions must be made through me. Choose Deck D”). If they did not obey, then they were instructed, “Always do as I say,” and were required to repeat that trial until they complied. These manipulations were designed to simulate freedom-granting (i.e., autonomy-supportive) versus coercive leaders [cf. 35,60]. The procedural fairness and self-determination individuals derive from such social and institutional decision situations is thought to be the primary source of procedural utility underlying freedom of choice [8,13].

Participants reported their outcome satisfaction after each trial, immediately after selecting a deck and viewing the card’s monetary value ($1 to $9). They rated their satisfaction on an 11-point scale, ranging from 1 (*very dissatisfied*) to 11 (*very satisfied*), with a neutral point at 6 (computer keys “1” to “-”, at the top of the keyboard, were relabeled).

#### Payoffs

During each trial, participants could receive a monetary payoff between $1 and $9. From an methodological standpoint, the payoff system was designed to create an internally consistent payoff scheme, as well as ensure that most participants would outperform the No-Choice Manager in the easy task, earning overall gains, but underperform compared to the No-Choice Manager in the harder task, earning overall losses. The precise methods for accomplishing these goals are described in detail in the Supporting Information (see *S1*.*2 Technical Design Elements* in S1 File). Here, we discuss key elements of the design: basic payoff scheme, expected payoffs associated with each manager.

There were 324 trials across the entire study: 162 trials per task (easier, harder), divided evenly between choice and no-choice (81 trials each). The number of trials was designed based on DeCaro et al. (2020) [13] and pilot testing to familiarize participants with each manager and give participants adequate time to learn the optimal deck in the easy task, and potentially learn the correct sequence in the hard task.

For comparison across choice procedures (choice, no-choice) and tasks (easy, hard), we ensured that participants received a specific minimum set of common outcomes across choice and no-choice trials in both the easy and hard tasks. Specifically, 27 trials (33%) were designed so that each payoff $1 to $9 was presented at random, an average of three times per condition (choice, no-choice), regardless of the deck chosen. This design ensured that we had at least an average of three observations per payoff $1 to $9, regardless of participants’ skill level (some individuals may otherwise perform especially well or poorly, limiting the range of payoffs observed). We used these trials as *diagnostic outcome-satisfaction trials* to compare outcome satisfaction for each payoff $1 to $9 across choice and no-choice conditions. Pilot testing revealed that individuals perceived these “random” diagnostic trials as variability or “noise” in the payoff distribution of the decks, enhancing the immersive experience of the task, which was designed to simulate a probabilistic system. Payoffs from the remaining 54 trials (67%) came directly from the underlying payoff distribution associated with the chosen deck (easy task) or sequence (hard task), as described earlier.

The No-Choice Manager was designed to perform moderately well, earning $5.71 (a slight gain) in both tasks. During the easy task, the No-Choice Manager required participants to choose the best deck (Deck D) 56% of the time, and the second-best deck (Deck B) 22% of the time, for an overall accuracy of 78% (earning $5.71). In the hard task, the No-Choice Manager instructed selection of correct deck sequence 78% of the time (earning $5.71). Thus, participants were presented with a moderately effective coercive manager, who was likely to perform better and earn more than participants on the hard task, but not the easy task. This design created a situation where participants may wish to sacrifice decision control to the comparatively more efficacious decision-maker in the hard task in exchange for greater economic security.

During Choice Manager trials, participants’ payoffs depend on their own decisions, contingent on their ability (efficacy) to learn. In the easy task, if a participant chooses completely at random (i.e., distributed evenly 25% across the four decks), then they will earn approximately $4.52, which is a loss that falls just below the $5 status quo (and $1.19 less than the *No-Choice Manager’s* $5.71). In contrast, if the participant chooses the optimal deck (Deck D) every trial, then they will earn approximately $7.08, which is a gain (and $1.37 greater than the *No-Choice Manager’s* $5.71). In DeCaro et al.’s (2020) experiment, using a similar task, most participants learned the optimal deck after about a third of the trials, achieving high accuracy thereafter. We therefore expected participants’ performance to fall between these extremes, earning approximately $6.02 on the easier task and better than the No-Choice Manager (see Supporting Information). Based on prior sequence learning studies (e.g., Raw et al., 2019), we expected most participants to perform poorly on the hard task, performing close to random. Random performance on the harder task generates approximately $3.53. Therefore, for the hard task, we expected most participants to perform worse than the No-Choice Manager, overall earning losses (approximately $3.53) compared to the No-Choice Manager’s gains (i.e., $5.71).

### Preference task

**Fig 2:**
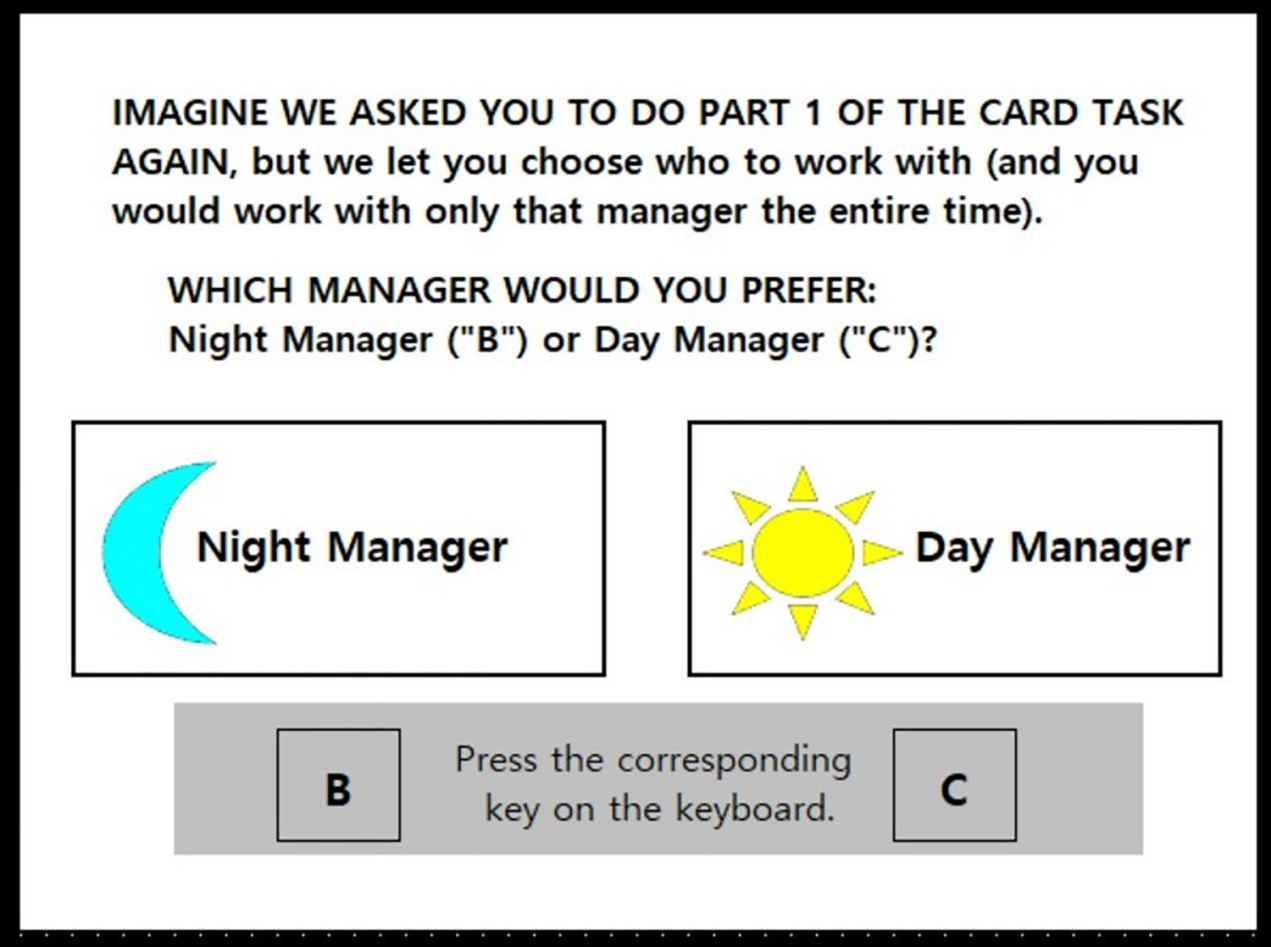
Example screenshot of the preference task. This example illustrates the preference question shown after Part 2 of the card task (easy/hard depends on order).

To assess preference for choice after each task (easy, hard), we asked participants who they would prefer to work with if they did that task again: Night Manager or Day Manager (Fig 2). In addition, we separately asked participants to indicate how strongly they would prefer, or not prefer, to work with each manager, on an 11-point scale (1 *Strongly prefer NOT TO work with this manager* to 11 *Strongly prefer TO work with this manager*). We also asked participants how satisfied they would be working with each manager, on an 11-point scale, (1 *Very Dissatisfied* to 11 *Very Satisfied*). We averaged responses to the latter two items into a single *preference strength* indicator (α = .84). We expected both preference (i.e., percentage choosing choice/no-choice) and preference strength to be similar, providing two indicators of preference for choice for greater validity.

### Follow-up measures

Participants completed several follow-up measures after each part of the card task (easier, harder). These measures consisted of manipulation checks, assessing participants’ perceptions of the task and each manager, in addition to their need satisfaction (i.e., procedural justice, self-determination, and security), thought to capture important elements of procedural utility and economic utility. Items were presented individually on separate screens. The *optimal response* question was presented first; the other items were presented in random order.

#### Optimal response

As an additional manipulation check, we asked participants whether they had learned the optimal response for each task. For the easy task, participants were asked to identify which deck (A, B, C, or D) yielded the highest payoffs, on average. For the harder task, participants were asked to input the correct sequence, using the same keys they used during the actual task.

#### Task difficulty

To assess perceived task difficulty, we asked participants, “How difficult was the card task that you just completed?” on an 11-point scale (1 *Very easy* to 11 *Very difficult*).

#### Self-efficacy

We used two items (*α* = .84) to assess participants’ perceived self-efficacy (e.g., “I was able to do a good job when I made decisions myself”). Participants responded on an 11-point scale (1 *Strongly Disagree* to 11 *Strongly Agree*). The items focused on personal efficacy when making decisions themselves, rather than across the entire study, because this was the critical aspect that participants controlled [61,62].

#### Perceptions of the managers

Items measuring perceptions of the managers began with the prompt, “When I worked with this manager…”). The managers were identified using the same name and symbol used throughout the card task. Items were presented in random order. Responses were reported on 11-point scales (1 *Strongly Disagree* to 11 *Strongly Agree*). We measured three critical aspects of each manager: the perceived procedural justice and self-determination they provided, their perceived efficacy, and the perceived security they granted.

*Procedural Justice and Self-Determination (PJSD)*. Procedural utility is thought to be derived from the satisfaction of two fundamental psychosocial needs associated with freedom of choice: *procedural justice* (i.e., fair decision processes) and *self-determination* (i.e., personal agency) [5,8,13,63]. We assessed three aspects of procedural justice (α = 0.89): *procedural fairness* (2 items; e.g., “I felt like the manager used a fair process to manage the decision situation”), *interpersonal justice* (2 items; e.g., “I felt like I was treated politely”), and *general fairness* (2 items; e.g., “I felt like I was treated justly”) [63,64]. We assessed two aspects of self-determination (α = 0.84): *self-concordance* (2 items; e.g., “I felt free to live life according to my desires”) [65] and *agency* (2 items; e.g., “My deck selections were determined by my own actions”) [66]. We combined these measures into a single procedural justice/self-determination (PJSD) factor (α = .91).

*Efficacy*. We used four items to assess two aspects of the managers’ efficacy (α = .93): *earning potential* (two items: e.g., “I was confident the manager and I would get good payoffs”) and *accuracy* (e.g., two items: “I was confident the manager and I would make accurate decisions”). We asked these questions referring to both the manager and the individual, because participants’ performance was contingent on both the manager’s behavior and the participants’ own decisions. For the No-Choice manager, this relationship is obvious: the manager controlled each decision, marginalizing individuals’ choice. Thus, the No-Choice Manager was directly responsible for any decision outcomes earned during no-choice trials, directly implicating their efficacy. For the Choice manager, this relationship was indirect, but still important. Specifically, even though participants selected which deck(s) to choose during choice trials, they were granted the authority to do so by the Choice Manager. Thus, the Choice Manager is responsible for allowing the participant to make decisions themselves, even in the hard task where such decision control is designed to yield worse outcomes and may be unwise.

*Security*. We used three items (α = .78) to assess how secure participants felt when working with each manager (e.g., “I felt safe from uncertainties”) [67].

#### Statistical methods and analyses

The research design, procedures, and materials were reviewed and approved by the University’s human subjects institutional review board to ensure ethical treatment of participants and their data (IRB #20.0039). Sample size was determined using conventions based on the sample sizes used in DeCaro et al.’s (2020) [13] prior experiment and the assumption of moderate/large effect sizes. From this information it was determined that approximately 60–80 participants would be sufficient to test the hypothesized effects. A total of 82 individuals participated. However, data from three individuals were excluded because they indicated during debriefing that they did not want to be paid, suggesting that economic incentives were perceived differently by these individuals. Two additional individuals were excluded because they failed to learn the optimal deck during the easier task, resulting in the final sample of 77 individuals. Exclusion of the individuals from the analyses did not alter the research findings or conclusions.

To test our hypotheses, we first report the results of the manipulation checks. These analyses were used to verify whether our manipulation of task difficulty and choice had the intended effects, creating a sense of difficulty, low self-efficacy, and insecurity in the hard task, as well as freedom of choice (Choice Manager) or coercion (No-Choice Manager). We examined the effect of task difficulty (easy, hard) and decision control (choice, no-choice) on performance (percent accuracy), perceived difficulty, and self-efficacy. We also report perceptions of the managers, examining perceived PJSD, efficacy, and security.

We then tested whether there was a utility bonus associated with choice, that was weaker for losses. To do so, we report outcome satisfaction as a function of task difficulty (easy, hard), choice (choice, no-choice), and outcome valence (losses, gains). Afterward, we report strength and percentage preference for choice (i.e., the Choice Manager).

The analyses were all conducted using standard significance tests (*α* < .05), using factorial ANOVAs. We used planned comparisons to probe the a-priori hypothesized interactions [68]. We included order effects (easy-hard, hard-easy) in preliminary analyses. The overall pattern of findings was the same. Therefore, we report the focal effects of task difficulty (easy, hard), manager (Choice, No-Choice), and outcome valence (losses, gains) here (order effects are reported in the S1 and S2 Figs in S1 File).

Next, we examine whether observed changes in preference for choice in the easy versus hard task were mediated by corresponding changes in perceptions of security. This analysis was conducted using Montoya and Hayes’s (2017) [69] method (*MEMORE 2*.*1*). Finally, we report the results of our computational cognitive modeling. The models were generated and tested using *MATLAB R 2020a*. All other analyses were conducted using *SPSS 28*.*01*. Given the complexity of the analyses, we provide more detail of the statistical methods throughout the results section.

## Results

### Manipulation checks

#### Easy versus hard task

*Performance*. Performance on the card task was measured by the percentage of correct decisions (e.g., choosing Deck D in the easy task) during Choice trials. We analyze performance during Choice trials, because that is when participants had control (the No-Choice Manager chose correctly 56% of the time in both tasks by design). As expected, participants performed substantially worse on the hard task, getting approximately 24% of the decisions correct (*M* = 24.10%, *95%CI*[22.86%,25.34%]), versus 73% correct in the easy task (*M* = 73.43%, *95%CI*[68.48%,78.39%]), *F*(1,75) = 321.13, *р* < .001, η_*p*_^2^ = .81. Furthermore, 100% of participants were able to state the optimal response (“Deck D”) for the easy task, and 0% were able to correctly enter the full, 9-step sequence for the hard task when prompted to do so. These patterns are consistent with participants learning the optimal response during the easy task, but failing to learn the optimal response in the hard task and, therefore, choosing at random.

As planned, participants outperformed the No-Choice Manager during the easy task (*t(76*) = 4.66, *p* < .001, *d* = 0.53), earning approximately $6.13 (*95%CI* [$5.95,$6.32]), which is a gain (above the $5 reference point). In contrast, most participants performed worse than the No-Choice Manager during the hard task (*t(76*) = -80.49, *p* < .001, *d* = -9.17), earning approximately $3.84 (*95%CI* [$3.79,$3.88]), which is a loss.

*Difficulty*. As expected, participants also perceived the hard task as more difficult (*M* = 6.87, *95%CI* [6.33,7.41]) than the easy task (*M* = 5.15, *95%CI* [4.58,5.72]), *F*(1,75) = 22.66, *р* < .001, η_*p*_^2^ = .23. Thus, our manipulation of task difficulty was successful, creating a difficult task in which participants performed worse than the No-Choice Manager and earned net losses.

#### Procedural justice and self-determination (PJSD)

Perceived PJSD was overall higher during the easy task (easy: *M* = 6.22, *95%CI*[6.01, 6.42]; hard: *M* = 5.86, *95%CI*[5.63, 6.10]), *F*(1,75) = 91.50, *р* < .003, η_*p*_^2^ = .11. As expected, participants perceived the Choice Manager as providing greater PJSD (*M* = 8.39, *95%CI*[8.02, 8.76]) than the No-Choice Manager (*M* = 3.69, *95%CI*[3.27, 4.12]), *F*(1,75) = 177.75, *р* < .001, η_*p*_^2^ = .70. The No-Choice Manager’s perceived PJSD was below the neutral point (“6”) of the scale (see *95%CI*), indicating that participants perceived the No-Choice Manager as coercive. This effect was qualified by a difficulty × manager interaction, *F*(1,75) = 11.75, *р* < .001, η_*p*_^2^ = .14. Participants perceived the Choice Manager as lower in PJSD in the hard task (easy: *M* = 8.83, *95%CI*[8.45, 9.21]; hard: *M* = 7.95, *95%CI*[7.47, 8.43]), *F*(1,75) = 16.19, *р* < .001, η_*p*_^2^ = .18. This result is consistent with a slight decrease in desire for choice in the hard task [11–12]. There was no difference for the No-Choice Manager (easy: *M* = 3.60, *95%CI*[3.11, 4.09]; hard: *M* = 3.78, *95%CI*[3.36, 4.20]). *F*(1,75) = 1.16, *р* = .284, η_*p*_^2^ = .02. The latter result is consistent with a floor effect: the coercive No-Choice Manager continues to be perceived as coercive (i.e., low in procedural justice and self-determination).

#### Efficacy

Our theory proposes that individuals must perceive an authority figure as more efficacious than themselves to be motivated to give decision control to that person. We therefore examined participants’ self-efficacy, the perceived efficacy of each manager, and the relative efficacy of the No-Choice manager compared to the participant, as a function of task difficulty.

*Self-Efficacy*. As expected, participants felt more efficacious during the easy task (*M* = 7.64, *95%CI* [7.25,8.03]) than the harder task (*M* = 4.73, *95%CI* [4.22,5.24]), *F*(1,75) = 86.68, *р* < .001, η_*p*_^2^ = .54. Participants’ self-efficacy in the hard task fell significantly below the neutral point (“6”) of the response scale (see *95%CI*), indicating that participants felt inefficacious at the hard task.

**Fig 3:**
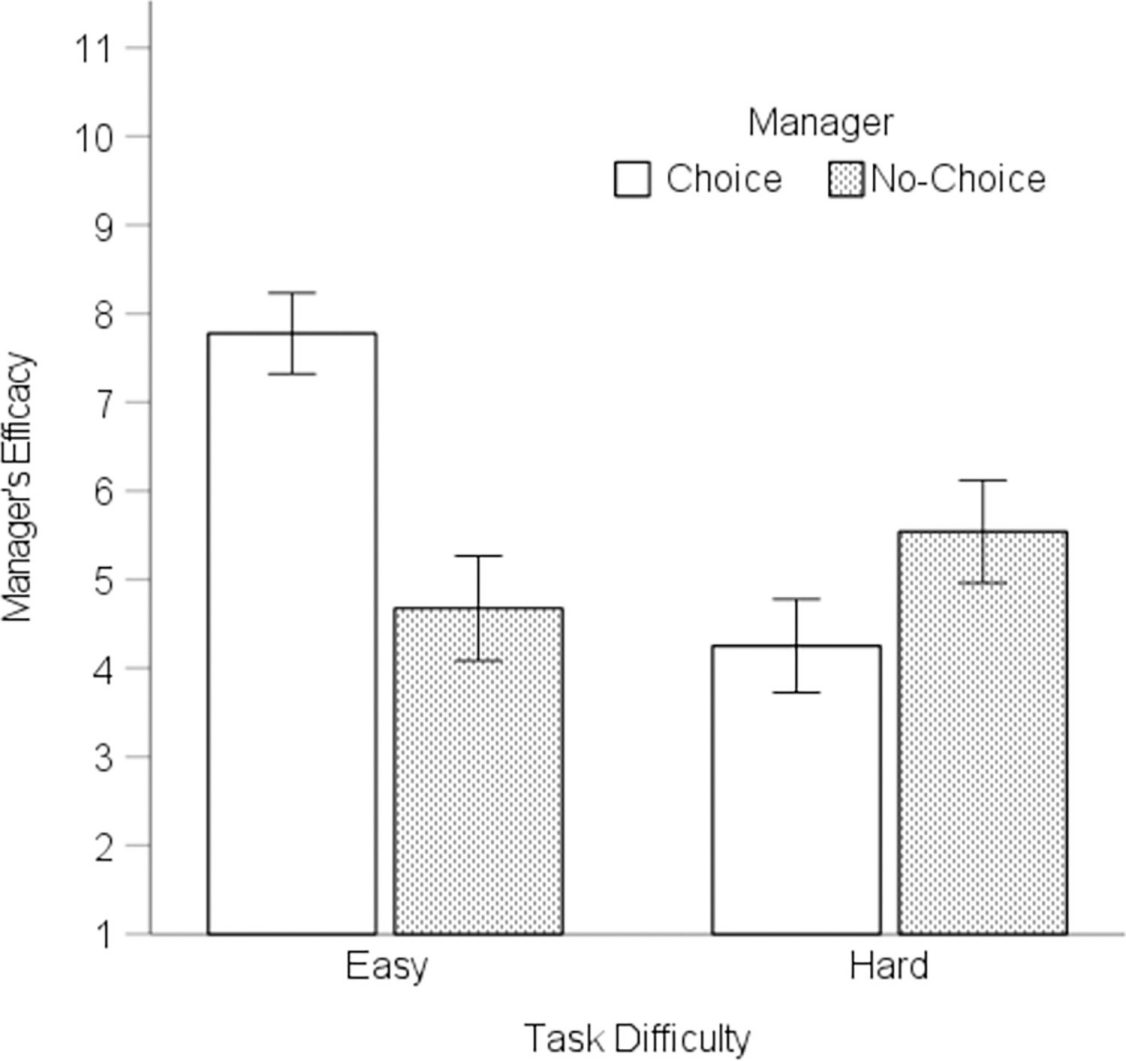
Perceived efficacy of the managers. Error bars = 1*SE*.

*Manager Efficacy*. To examine the perceived efficacy of the managers, we conducted a 2 (difficulty: easy, hard) × 2 (manager: choice, no-choice) within-subjects factorial ANOVA. Overall, participants perceived that the Choice Manager (*M* = 6.01, *95%CI* [5.65,6.38]), which granted participants full decision control, was more efficacious than the No-Choice Manager (*M* = 5.11, *95%CI* [4.64,5.58]), which controlled each decision, *F*(1,75) = 9.31, *р* = .003, η_*p*_^2^ = .11. This effect were qualified by a difficulty × manager interaction (Fig 3), *F*(1,75) = 65.64, *р* < .001, η_*p*_^2^ = .47. During the easy task, participants perceived the Choice Manager as more efficacious (*M* = 7.78, *95%CI* [7.32,8.23]) than the No-Choice manager (*M* = 4.68, *95%CI* [4.09,5.27]), *F*(1,75) = 53.77, *p* < .001, η_*p*_^2^ = .42. This pattern reversed in the hard task. During the hard task, participants perceived the No-Choice Manager (*M* = 5.54, *95%CI* [4.96,6.12]) as more efficacious than the Choice Manager (*M* = 4.25, *95%CI* [3.73,4.78]), *F*(1,75) = 11.51, *p* = .001, η_*p*_^2^ = .13.

*Relative Efficacy*. To compare how participants perceived their self-efficacy relative to the No-Choice Manager, we computed a difference score (*Self-Efficacy*–*No-Choice Manager’s Efficacy*). As anticipated, participants felt more efficacious than the No-Choice Manager during the easy task (*M* = 2.97, *95%CI* [2.24, 3.70], but less efficacious than the No-Choice Manager during the hard task (*M* = -0.81, *95%CI* [-1.44, -0.19]), *F*(1,75) = 65.23, *р* < .001, η_*p*_^2^ = .47.

#### Security

To examine the perceived security associated with each manager, we conducted a 2 (difficulty: easy, hard) × 2 manager (choice, no-choice) within-subjects factorial ANOVA. Overall, participants felt more secure during the easy task (*M* = 6.13, *95%CI* [5.84,6.42]) than the harder task (*M* = 4.90, *95%CI* [4.51,5.28]), *F*(1,75) = 44.94, *р* < .001, η_*p*_^2^ = .38. They also felt more secure making decisions themselves (choice: *M* = 5.89, *95%CI* [5.49,6.28]; no-choice: *M* = 5.14, *95%CI* [4.66,5.62]), *F*(1,75) = 4.89, *р* = .030, η_*p*_^2^ = .06. These effects were qualified by a difficulty × manager interaction, *F*(1,75) = 37.72, *р* < .001, η_*p*_^2^ = .34. During the easy task, participants felt more secure making decisions themselves (choice: *M* = 7.27, *95%CI* [6.79, 7.74]; no-choice: *M* = 5.00, *95%CI* [4.46,5.54]), *F*(1,75) = 41.80, *p* < .001, η_*p*_^2^ = .56. During the hard task, they felt more secure when the No-Choice Manager dictated their decisions (choice: *M* = 4.51, *95%CI* [3.96,5.06]; no-choice: *M* = 5.29, *95%CI* [4.70,5.87]), *F*(1,75) = 4.93, *p* = .029, η_*p*_^2^ = .04.

### Outcome satisfaction

#### Losses

To determine whether participants indeed perceived the outcomes $1 through $4 as losses, we quantified the extent to which outcome satisfaction ratings for losses and gains deviated from “6,” the neutral point, in both relative and absolute value. As expected, participants rated losses negatively (*M* = -3.41, *95%CI* [-3.69, -3.13]) and gains positively (*M* = 2.32, *95%CI* [2.08, 2.86]), *F*(1,75) = 772.20, *p* < .001, η_p_^2^ = .91. Participants also reacted more intensely to losses than gains: the average, absolute deviation was greater for losses (*M* = 3.48, *95%CI* [3.24, 3.72]) than gains (*M* = 2.38, *95%CI* [2.17, 2.59]), *F*(1,75) = 62.91, *p* < .001, η_p_^2^ = .46. These findings affirm our manipulation of the outcomes $1 through $4 as losses, and $6 through $9 as gains.

#### Satisfaction curves

**Fig 4:**
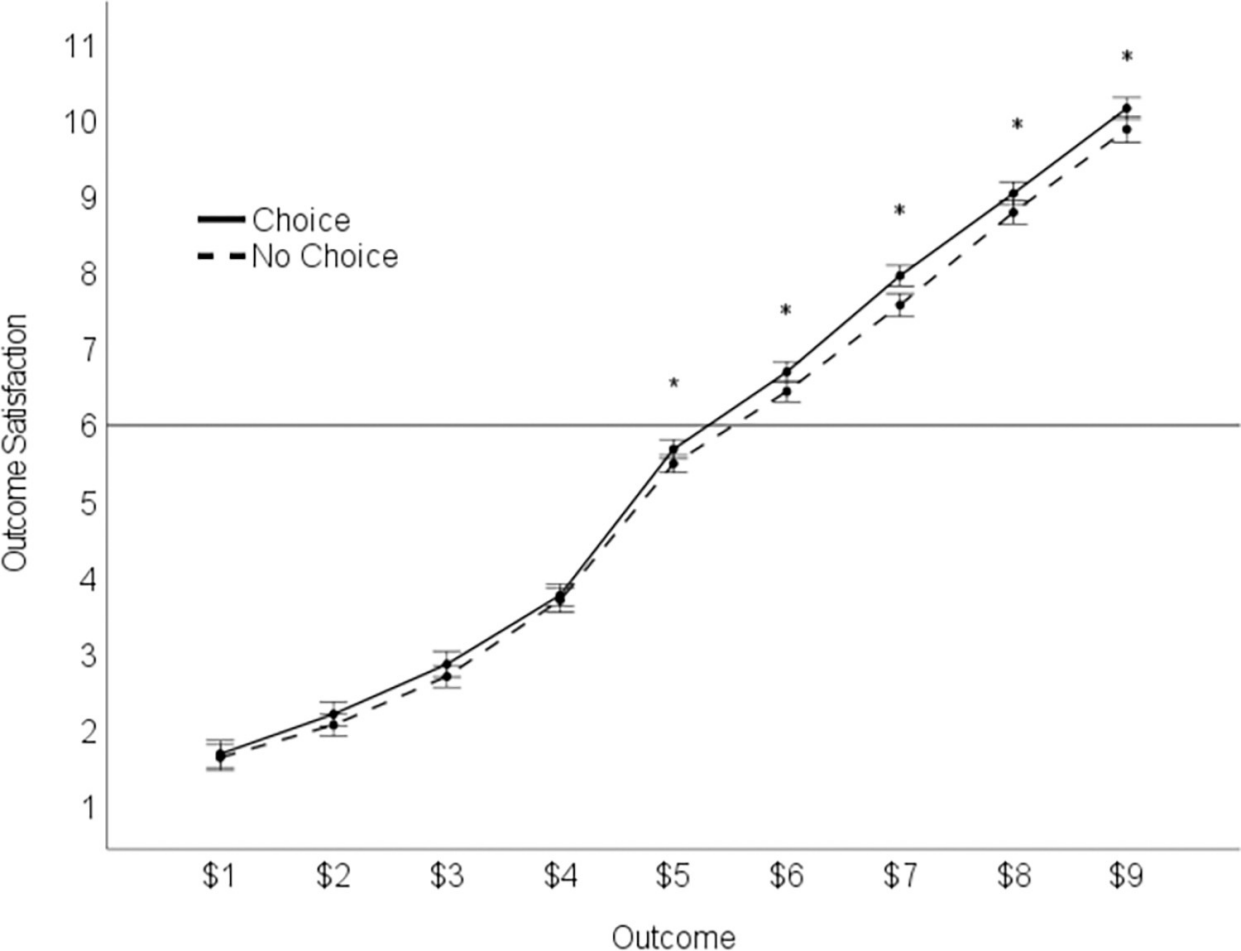
Mean outcome satisfaction for each payoff as a function of choice. Error bars = 1*SE*. *р < .05 (see S5 Table in S1 File).

An asymmetric, *S-*shaped value function is often used to conceptualize the greater impact (disutility) of losses compared to equally-sized gains, under strict assumptions of loss aversion [57]. However, negativity bias requires only an overall disutility (dissatisfaction) for losses, with no particular underlying value function or curve [33]. As shown in Fig 4, the pattern of outcome satisfaction imperfectly resembled an asymmetric, *S*-shaped curve. Specifically, outcome-satisfaction for the $5 status quo outcome, representing the division between losses and gains, was slightly negative (*M* = 5.59, *95%CI* [5.36, 5.82]), falling below the neutral point “6.” However, as reported earlier, losses were, on average, felt more negatively and extremely than equally-sized gains. Gains exhibited a steady (rather than diminishing) positive utility (i.e., each additional dollar earned above $5 added a consistent amount to outcome satisfaction). This pattern is consistent with both negativity bias [33] and gains-seeking [70]. Individuals were motivated to avoid losses and approach gains.

#### Additional utility of choice

**Fig 5:**
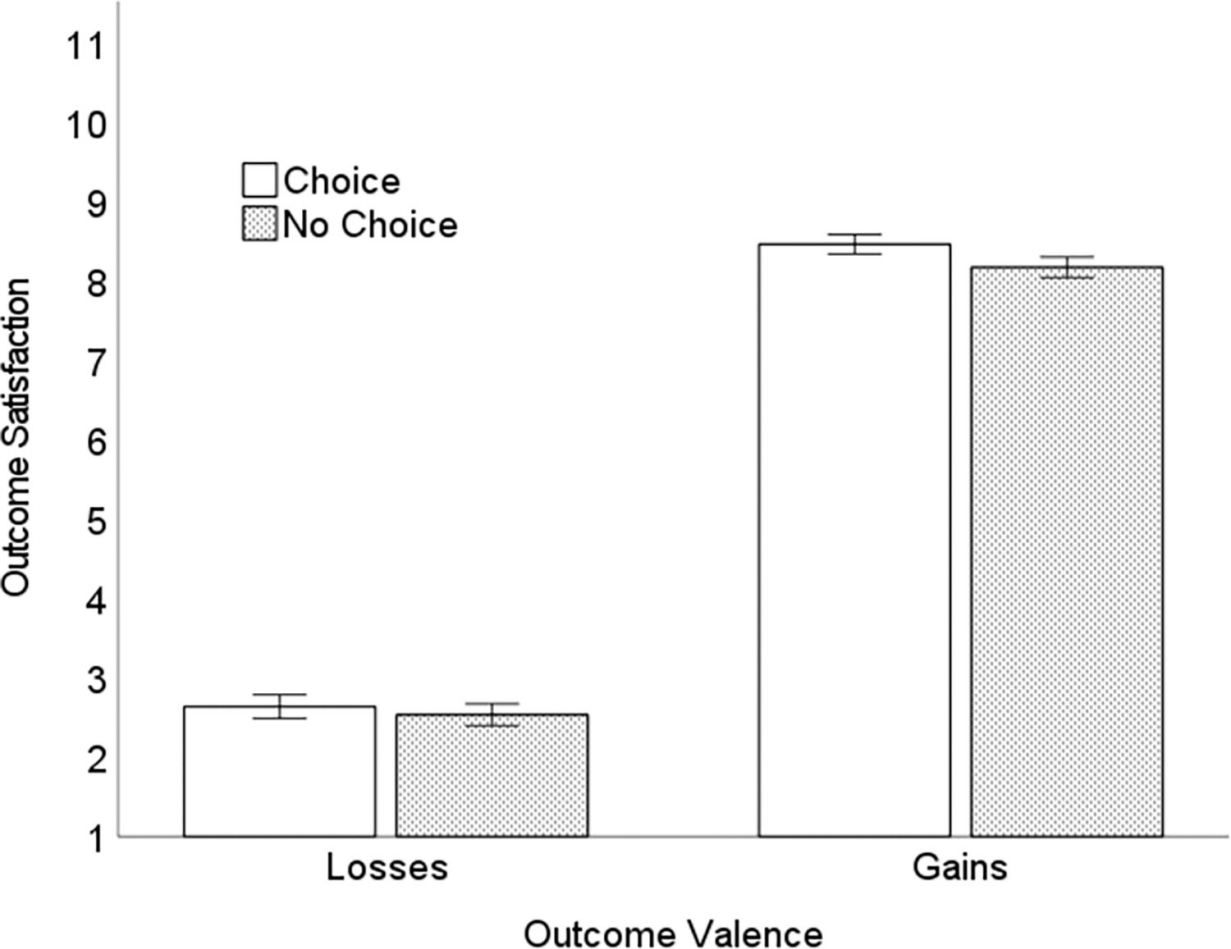
Mean outcome satisfaction for losses versus gains as a function of choice. Error bars = 1*SE*.

To test whether individuals, indeed, experience greater satisfaction (“utility”) for outcomes earned by choice, and to test whether this beneficial effect of choice decreases for losses (i.e., $1 to $4), we conducted a 2 (difficulty: easy, hard) × 2 (manager: choice, no-choice) × 2 (outcome valence: losses, gains) factorial ANOVA. There was a main effect of outcome valence, *F*(1,75) = 772.20, *p* < .001, η_p_^2^ = .91. Participants derived less satisfaction from losses (*M* = 2.59, *95%CI* [2.31, 2.87]) than gains (*M* = 8.32, *95%CI* [8.08, 8.56]). There was a main effect of choice, *F*(1,75) = 7.92, *p* = .006, η_p_^2^ = .10. Overall, participants were more satisfied by payoffs earned via choice, with the Choice Manager (*M* = 5.55, *95%CI* [5.38, 5.73]), than via no-choice with the No-Choice Manager (*M* = 5.36, *95%CI* [5.18, 5.53]). Overall, satisfaction did not differ by difficulty (easy: *M* = 5.45, *95%CI* [5.25, 5.65]; hard: *M* = 5.46, *95%CI* [5.29, 5.62]), *F*<1, *p* = .925, η_p_^2^ = .00). However, as expected, these effects were qualified by a manager × outcome valence interaction, *F*(1,75) = 8.45, *p* = .005, η_p_^2^ = .10 (Fig 5). Choice resulted in greater outcome satisfaction (*M* = 8.47, *95%CI* [8.22, 8.71]) than No-Choice (*M* = 8.18, *95%CI* [7.91, 8.44]) when the outcomes were gains, *F*(1,75) = 13.39, *p* < .001, η_p_^2^ = 0.15. Choice did not result in greater outcome satisfaction when the outcomes were losses (choice: *M* = 2.64, *95%CI* [2.34, 2.94]; no-choice: *M* = 2.55, *95%CI* [2.26, 2.81]), *F*(1,75) = 1.88, *p* = .174, η_p_^2^ = .024. There was no 3-way interaction, *F*(1,75) = 0.05, *p* = .599, η_p_^2^ = .004. Thus, this pattern did not differ in the easy versus hard task environment. Specifically, the additional satisfaction individuals derived from Choice emerged only for gains, in both the easy and hard tasks.

### Preference for choice

Our outcome of primary interest is preference for choice. We separately examined preference strength and percentage preference for the Choice Manager (i.e., choice). For the easy task, we expected participants to prefer the No-Choice Manager more strongly than the No-Choice Manager and exhibit greater percentage preference for Choice. We expected the opposite for the hard task.

#### Preference strength

**Fig 6:**
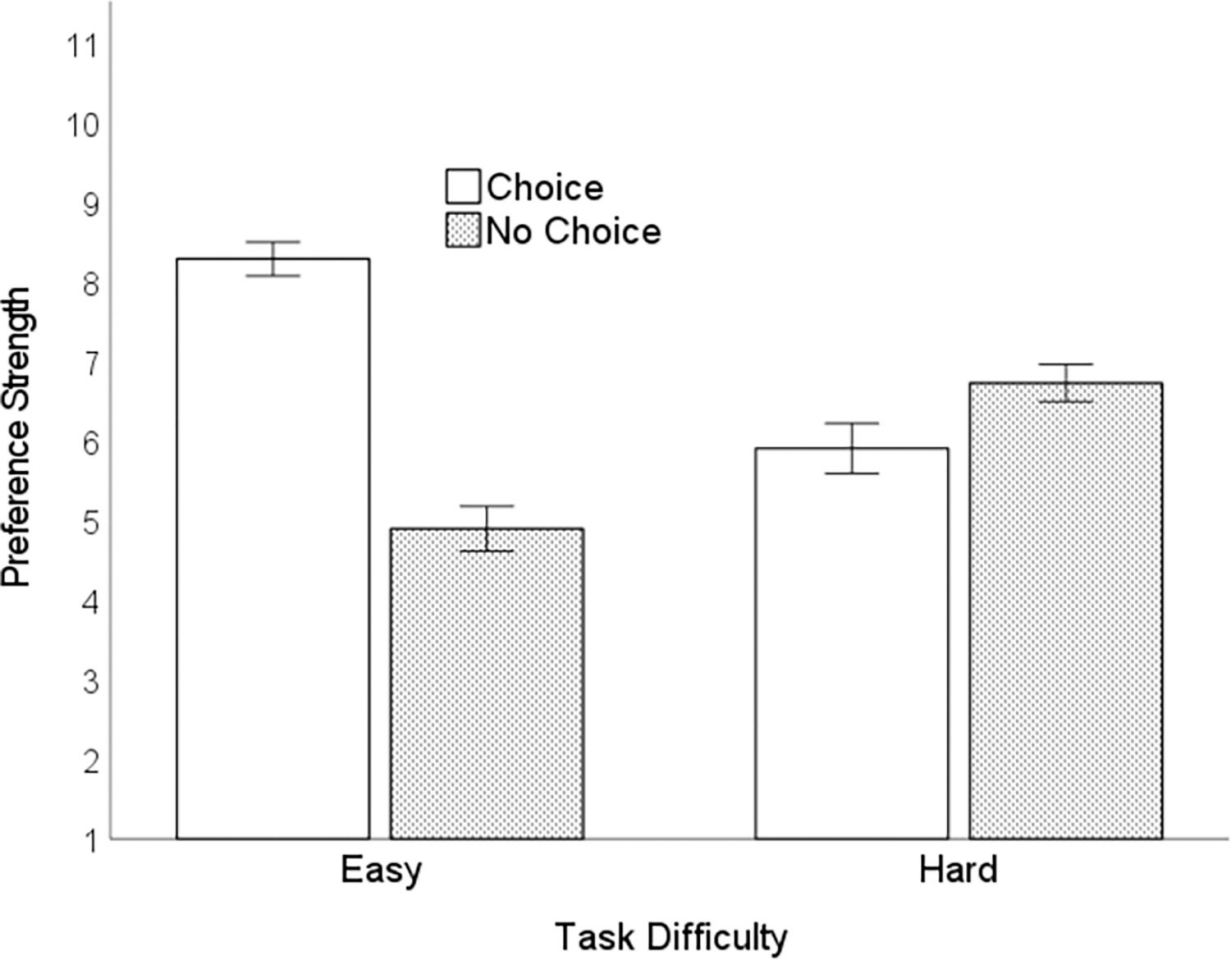
Strength of preference for the choice versus no-choice manager. Error bars = 1*SE*.

To examine preference strength, we used a 2 (difficulty: easy, hard) × 2 (manager: choice, no-choice) repeated-measures ANOVA. There was a main effect of manager, *F*(1,75) = 12.74, *p* = .001, η_p_^2^ = .15. On average, participants preferred the Choice Manager (i.e., having freedom of choice) more strongly (*M* = 7.11, *95%CI* [6.70, 7.52]) than the No-Choice Manager (*M* = 5.83, *95%CI* [5.42, 6.23]). There was a main effect of difficulty: preferences were stronger, overall, in the easy task (*M* = 6.61, *95%CI* [6.42, 6.80]), versus hard task (*M* = 6.32, *95%CI* [6.04, 6.60]), *F*(1,75) = 4.21, *p* = .044, η_p_^2^ = .05. This effect indicates that individuals exhibited stronger preferences for both (i.e., any) manager when the task was easier. As expected, these effects were qualified by the focal difficulty × manager interaction, *F*(1, 75) = 52.73, *p* < .001, *η*^*2*^ = .41 (Fig 6), which tests differences in strength of preference for the Choice versus No-Choice Manager in the easy versus hard task. During the easy task, participants preferred the Choice Manager (*M* = 8.32, *95%CI* [7.90, 8.74]) more strongly than the No-Choice Manager (*M* = 4.90, *95%CI* [4.35, 5.46]), *F*(1,75) = 67.51, *p* < .001, η_p_^2^ = .47. This preference reversed in the hard task: participants preferred the No-Choice Manager (*M* = 6.75, *95%CI* [6.29, 7.21]) more strongly than the Choice Manager (*M* = 5.90, *95%CI* [5.28, 6.51]), *F*(1,75) = 4.21, *p* < .044, η_p_^2^ = .05. The strength of preference for the Choice Manager in the easy task (η_p_^2^ = .47) was stronger than the strength of preference for the No-Choice Manager in the hard task (η_p_^2^ = .05). This finding likely indicates that individuals were not enthusiastic about choosing the No-Choice Manager in the hard task, though many participants did, as demonstrated in the analysis of percentage preference for choice.

#### Percentage preference for choice

To examine percentage preference for choice, we calculated the number of participants who selected the Choice Manager after the easy versus hard task. As anticipated, a greater number of participants preferred the Choice Manager when the task was easy (*M* = 84.49%, *95%CI* [76.16%, 92.83%]) compared to hard (*M* = 43.48%, *95%CI* [32.70%, 54.26%]), *F*(1,75) = 35.66, *p* < .001, η_p_^2^ = .32. This result translates into an approximately 41% decrease in preference for Choice in the hard task.

We also ran a series of follow-up tests to investigate potential differences in preference for the Choice Manager associated with participant gender, education, and race. There were no significant effects of any factor. We report these results for clarification.

Fifty-six individuals identified as female, 20 as male, and 1 as non-binary. To examine potential effects associated with gender, we included gender (female, male) in the above analyses. We excluded the one individual who identified as non-binary due to insufficient sample size (including this individual does not affect the results). There was no main effect of gender (female: *M* = 66.07%, *95%CI* [57.77%, 74.37%]; male: *M* = 57.50%, *95%CI* [43.61%, 71.39%]), *F*(1,74) = 1.11, *p* = .295, η_p_^2^ = .02. There was no gender × difficulty interaction, easy (female: *M* = 85.71%, *95%CI* [75.90%, 95.53%]; male: *M* = 80.00%, *95%CI* [63.57%, 96.43%]), hard (female: *M* = 46.43%, *95%CI* [33.12%, 59.73%]; male: *M* = 35.00%, *95%CI* [12.74%, 57.26%]), *F*<1, *p* = .724, η_p_^2^ = .00.

There were 41 freshman, 20 sophomores, 9 juniors, and 7 seniors in the sample. There was no main effect of education (freshman: *M* = 65.85%, *95%CI* [56.05%, 75.65%]; sophomore: *M* = 62.50%, *95%CI* [48.47%, 76.53%]; junior: *M* = 55.56%, *95%CI* [34.64%, 76.47%]; senior: *M* = 64.29%, *95%CI* [40.57%, 88.00%]), *F*<1, *p* = .843, η_p_^2^ = .01. There was no education × difficulty interaction (Table 1), *F*<1, *p* = .985, η_p_^2^ = .00.

**Table 1:** Education and percentage preference choice (Easy vs. Hard).

Education	Easy	Hard
	*Mean [95CI]*	*Mean [95CI]*
Freshman	85.37% [73.80%, 96.93%]	46.34% [30.58%, 62.10%]
Sophomore	85.00% [68.44%, 100.00%]	40.00% [17.44%, 62.56%]
Junior	77.78% [53.09%, 100.00%]	33.33% [0.00%, 66.97%]
Senior	85.71% [57.72%, 100.00%]	42.86% [4.72%, 80.99%]

*N =* 74.

Forty-eight individuals identified as Caucasian, 10 African American, 6 Biracial, 5 Asian, 3 Hispanic, and 4 as “other.” There was insufficient sample size to conduct a detailed analysis based on each racial identification. Therefore, we organized racial identification into three categories (Caucasian, African American, and “All Others”). There was no main effect of race (Caucasian: *M* = 60.42%, *95%CI* [51.46%, 69.37%]; African American: *M* = 70.00%, *95%CI* [50.37%, 89.63%]; All Others: *M* = 66.67%, *95%CI* [52.04%, 81.30%]), *F*(2,73)<1, *p* = .584, η_p_^2^ = .02. There was no race × difficulty interaction (Table 2), *F*<1, *p* = .981, η_p_^2^ = .00.

**Table 2:** Race and percentage preference choice (Easy vs. Hard).

Education	Easy	Hard
	*Mean [95CI]*	*Mean [95CI]*
Caucasian	81.25% [70.61%, 91.89%]	39.58% [25.13%, 54.03%]
African American	90.00% [66.69%, 100.00%]	50.00% [18.34%, 81.66%]
All Others	88.89% [71.51%, 100.00%]	44.44% [20.85%, 68.04%]

*N =* 74.

### Mediation

Our general prediction was that the preference to give up decision-making control to a coercive authority figure is influenced by the relative security that authority can provide compared to oneself. If the manager can provide more security than the individual is able to provide when making their own decisions, then the individual should exhibit a preference reversal, relinquishing decisional control. Therefore, change in preference for choice from the easy to hard task should be mediated by corresponding changes in the relative security the No-Choice Manager provides.

To test this hypothesis, we first created a difference score (*Choice Security–No-Choice Security*), representing the security participants felt when making decisions themselves, with the Choice Manager, versus under the direct control of the No-Choice Manager. Positive difference scores represent greater security making decisions oneself, whereas negative scores represent greater security making decisions under the direct control of the No-Choice Manager. As expected, participants felt more secure making decisions themselves during the easy task (*M* = 2.27, *95%CI* [1.44, 3.09]), but felt more secure making decisions under the control of the No-Choice Manager during the hard task (*M* = -0.78, *95%CI* [-1.62, 0.06]), *F*(1,75) = 37.72, *p* < .001, η_p_^2^ = .34.

Next, we investigated whether the changes in relative security (*M*_*1*_: *R*.*SEC*) mediated the effect of task difficulty (*X*: *Easy*, *Hard*) on percentage preference for choice (*Y*: *P*.*Choice*). We quantified this indirect effect using *MEMORE 2*.*1* [69] and Hayes’ (2018) [71] index of mediation, with 10,000 bootstrapped samples for stable estimates. To maintain consistency with our previous analyses, we coded the underlying model to refer to percentage preference for choice as a function of task difficulty (easy *minus* hard).

**Fig 7:**
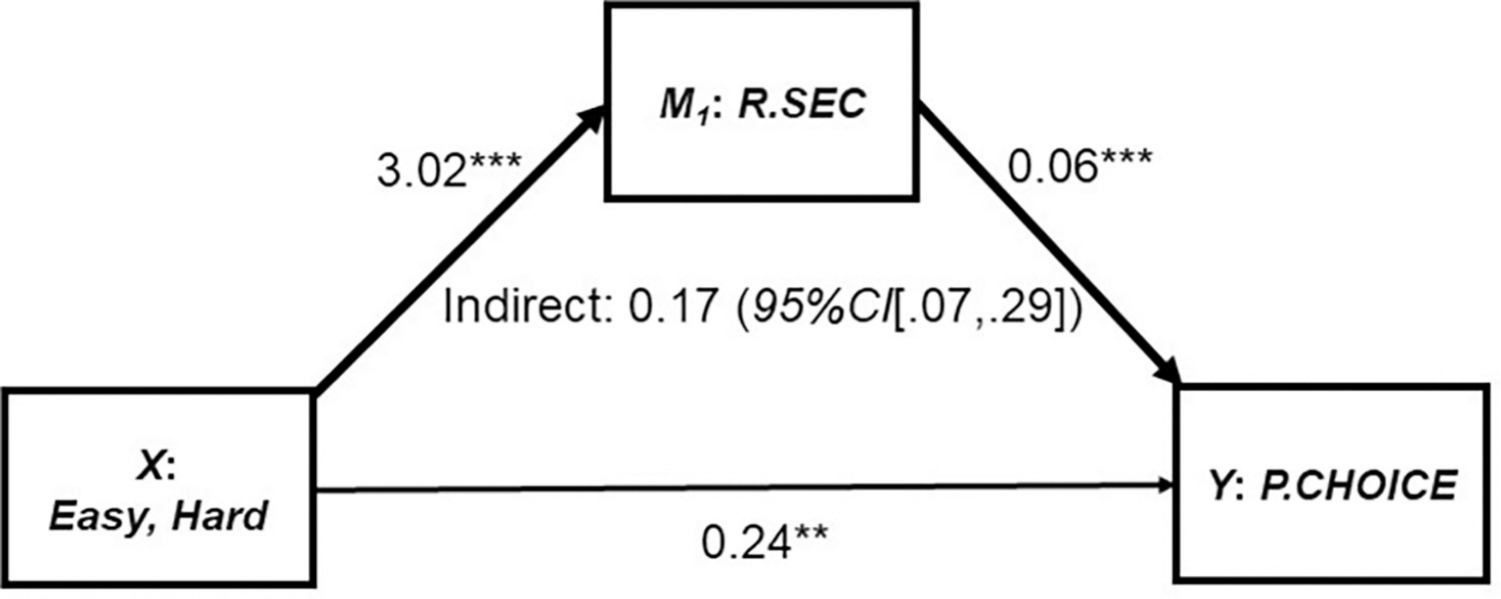
Mediation model: Percentage preference for choice. *M*_*1*_(R.SEC) = relative security (security difference score, Choice vs. No-Choice in the easy vs. hard task). *Y*: *P*.*CHOICE =* percentage preference for the No-Choice Manager. Indirect effect bolded. *N =* 77. **p < .01 ***p < .001.

The detailed results are reported in Table 3. The mediational model is illustrated in Fig 7. First, as previously demonstrated, participants felt more secure (*M*_*1*_: *R*.SEC) making decisions themselves during the easy task (*B =* 3.02, *p* < .001). Second, task difficulty (*X*), *B =* 0.24, *p* = .003, and relative security (*M*_*1*_: *R*.*SEC*), *B =* 0.06, *p <* .001, each had direct effects on percentage preference for the Choice Manager (*Y*: *P*.*CHOICE*). Specifically, when accounting for both task difficulty and relative security, preference for Choice was an average of 24% higher during the easy task. Participants were 6% more likely to prefer Choice with each 1-unit increase in perceived security.

**Table 3:** Model coefficients: Percentage preference for choice.

	M_1_: R.SEC			Y: P.CHOICE		
	Coeff(*SE*)	*p*	*95CI*	Coeff(*SE*)	*p*	*95CI*
*X* (E,H)	3.02(.50)	< .001	[2.03,4.01]	0.24(.08)	.003	[0.09,0.40]
*M*_*1*_(R.SEC)	—	—	—	0.06(.01)	< .001	[0.03,0.09]
*M*_*1*_(AVG)	—	—	—	-0.02(.02)	.341	[-0.06,0.02]
				*R*^*2*^ = .1748		
				*F*(2,74) = 7.48, *p* < .001		

*X* (E,H) = condition within-subjects (easy, hard). *M*_*1*_(R.SEC) = relative security (security difference score, Choice vs. No-Choice in the easy vs. hard task). *Y*: *P*.*CHOICE =* percentage preference for the No-Choice Manager. *M*_*1*_(AVG) = average security. *N =* 77.

Importantly, the hypothesized mediational pathway (i.e., indirect effect) linking task difficulty (*X*) to change in preference for Choice (*Y*: *P*.*CHOICE*), via corresponding changes to relative security (*M*_*1*_: *R*.*SEC*), was significant (*Index* = 0.17, *95%CI* [0.07,0.29]). Due to this pathway, preference for Choice increased by an additional 17% for each 1-unit increase in relative security, caused by the easy versus hard task. These results support our assumption that performance-contingent perceptions of security contribute to preferences for choice.

### Cognitive models

To better understand the potential cognitive processes involved in performance-contingent preference for Choice, we used Decision Field Theory’s computational cognitive modeling framework to develop and test three diagnostic models. *Decision Field Theory* (DFT) [32] has been used to describe a variety of decisions in many domains [72,73]. Building on DeCaro et al. (2020) [13], we hypothesized that attentional focus plays a key role in the decision calculus underlying preference for Choice. Specifically, financial losses, which threaten economic security, should capture attention. This attentional capture should lead individuals to focus primarily on economic aspects of Choice (i.e., outcome utility), rather than freedom of choice (i.e., procedural utility), when deciding whether or not to retain or abandon decision control. DFT is ideal for this analysis, because it uses a central, attention-based mechanism to determine the value, or utility, associated with decision options [74,75]. The current analysis represents a novel application of DFT to incorporate procedural utility. Therefore, we first explain the key characteristics of DFT, followed by our adaption of DFT to examine potential changes in the focus of attention, as a function of losses.

DFT is a *sequential sampling model*. Such models posit that choices are made by accumulating information over time, until preference strength for one option reaches a critical threshold [76]. According to DFT, individuals accumulate information by thinking about the characteristics and possible outcomes of each option: each moment, the decision-maker attends to a different characteristic (dimension) of the options, using the perceived strengths and weaknesses to update an ongoing preference state. Eventually, sufficient evidence accumulates in favor of one option, and a choice is made.

Consider the decision between the Choice and No-Choice managers. We assume that participants define these options along two dimensions. The *outcome utility* dimension represents the value derived from the monetary payoffs earned while working for each manager. For our models, we used participants’ final earnings in each of the card tasks (easy, hard), with the Choice versus No-Choice Manager. The *procedural utility* dimension refers to the value participants derive from exercising freedom of choice. We used participants’ ratings of PJSD associated with each manager (Choice, No-Choice), during the easy versus hard task, to determine this value.

These two dimensions (outcome vs. procedural utility) likely compete for attention and, therefore, influence over people’s preferences for choice. DFT posits that, at each moment in time, an individual attends to only one dimension, when comparing options. For example, an individual may first pay attention to the outcome utility dimension, considering the value they derive from the monetary payoffs associated with the Choice and No-Choice Managers. The next moment, they may focus attention to the procedural utility dimension, considering the value they derive from the PJSD of the Choice and No-Choice Managers. This information is updated over time, adjusting the overall value associated with each option, until a final selection is made. Individuals may also enter a decision situation with an initial preference, or bias, in favor of a particular option. For example, in Western democracies, individuals often have an initial preference for personal decision control [3,77]. As described next, DFT can account for each of these factors, using three core elements: a process for computing and updating the perceived value of each option, a bias parameter, and attention “weights.”

#### Computing value

The underlying utility calculations are computed as follows. At each moment in time, DFT computes a *valence*, *V*, which represents the current difference in value associated with the Choice Manager, *ν*_*C*_, versus the No-Choice Manager, *ν*_*NC*_:

$$V=\nu_{C}-\nu_{NC}$$ (1)

When attention is focused on procedural utility, *V* (current valence) is updated by comparing the value associated with Choice versus No-Choice. In our study, the Choice Manager was perceived as having greater PJSD (i.e., value) than the No-Choice Manager. Thus, *V* will typically be *V* > 0, adding to the overall positive value of Choice. When attention is focused on the outcome utility dimension, the update to *V* (current valence), will depend on the participants’ actual earnings. First consider the easy task. Most participants earned gains with the Choice Manager, and these gains were greater than the gains earned with the No-Choice manager. Therefore, when deciding which manager to work for in the easy task, *V* will be *V* > 0 for most participants, adding positively to the perceived value of Choice. Now consider the hard task. Most participants earned losses with the Choice Manager but earned gains with the No-Choice Manager. Therefore, *V* will be *V* < 0, adding positively to the perceived value of No-Choice.

A participant’s final decision to select Choice over No-Choice in the easy or hard task is the total of these momentary valences, at the moment of decision. In DFT, this updating process involves updating the *preference state P*(t) for a particular manager (option) at a particular time, *t*. The preference state is defined as the sum of the previous preference state, *P*(t-1), and the new valence, *V*:

P(t)=P(t−1)+V(t−1). (2)

#### Initial bias

DFT contains a *bias paramete*r, *ϐ*, which represents any potential initial bias in favor of a particular option. Specifically, at the beginning of deliberation, the *initial preference state*, *P*(0) = *ϐ*, can be used to account for an initial bias. As previously stated, individuals in Western democracies can be assumed to have an initial bias in favor of Choice. DFT allows the bias parameter to be treated as a free-parameter, estimating the degree of bias present in individuals’ decisions, if any. Thus, if *ϐ* > 0, then there is an initial bias in favor of the Choice Manager.

The bias parameter influences the final decision as follows. According to DFT, individuals consider each option until a sufficient amount of evidence is collected to push them beyond some *decision threshold*, *ϑ*. DFT assumes that *ϑ* represents a person’s level of caution, or stringency in making a decision. This factor is typically estimated from the observed decision data, without an a-priori reason to select a particular value up front. The bias parameter, *ϐ*, affects the decision by decreasing the amount of evidence/information needed to select the initially favored option (and increasing the information needed to select the disfavored option). In the current study, bias in favor of Choice means that less evidence is needed for an individual to reach the decision threshold, *ϑ*, and select the Choice Manager.

#### Attention “weights”

As stated earlier, we hypothesized that the final decision for/against Choice depends on task difficulty, and which dimension (procedural vs. outcome utility) individuals focus on in the easy versus hard task. Our theory assumes that, when deciding which manager to work for, individuals place more attention (“weight”) on the outcome utility dimension when facing a net loss (i.e., the hard task). DFT determines the focus of attention, based on an *attention parameter*, *w*. In our model, *w*, defines the probability that an individual attends to the outcome utility dimension, and 1-*w* is the probability of attending to the procedural utility dimension. Thus, *w* determines which dimension is currently being considered and, therefore, the momentary values of *ν*_*C*_ and *ν*_*NC*_ in the overall valuation process, *V*, noted earlier. We estimated separate attention weights for the easy versus hard tasks (*w*_*easy*_, *w*_*hard*_) to capture changes in the balance of attention, if any, associated with participants’ performance in each of those tasks.

#### Models tested

We created and tested three diagnostic models, meant to represent three dominant theoretical perspectives (Table 4). We treated the decision threshold (*ϑ*), bias parameter (*ϐ*), and attention weights (*w*_*easy*_, *w*_*hard*_), as free parameters, to determine the most likely value of those parameters to best describe participants’ decision behaviors in the preference task. *Model 1 (Null)* represents a standard economic utility model that ignores procedural utility. This model bases the valence of Choice entirely on the average economic outcomes (monetary payoffs) participants earned. Thus, it must predict preference for Choice in the easy task, if participants earned more money with the Choice Manager in the easy task. Conversely, Model 1 (Null) must predict preference for No-Choice in the hard task, if participants earned more money with the No-Choice Manager. *Model 2 (Biased)* adds the bias parameter (*ϐ*), representing a potential initial preference for Choice, similar to standard assumptions in procedural utility theory. *Model 3 (Full)* represents our hypothesis that decision-makers consider both economic outcomes (outcome utility) and decision procedures (procedural utility) but attend to these dimensions to different extents when facing losses (hard task) or gains (easy task).

**Table 4:** Cognitive model components.

Model	*ϐ*	*w*_easy_, *w*_hard_
1) Null		
2) Biased	x	
3) Full	x	x

*ϐ* = potential initial bias for Choice. *w*_easy_, *w*_hard_ = potential losses-dependent attentional weighting of outcomes/procedures for the easy vs. hard task.

#### Model estimation

We used the models to predict participants’ percentage preference for choice data (from the preference task). Prior to modeling, we standardized the underlying scales for monetary payoffs (i.e., $1:$9) and perceived PJSD (i.e., 1:11) to improve parameter interpretability. To do so, we followed the procedure use by Berkowitsch, et al. (2015) [78]. We rescaled attribute values (payoffs, PJSD ratings) to have the same range, from *min*_*new*_ = 0 to *max*_*new*_ = 1, according to:

$$\nu_{new}={min}_{new}+\frac{\left(\nu_{old}-{min}_{old})({max}_{new}-{min}_{new}\right)}{{max}_{old}-{min}_{old}},$$

Where min_old_ is the theoretical minimum value of the original attribute range (2.99 for payoffs and 1 for PJSD ratings) and max_old_ is the theoretical maximum value of the of the original attribute range (7.08 for payoffs and 11 for PJSD ratings).

#### Model performance

We evaluated each model’s performance in terms of its ability to replicate the overall (mean) preference patterns for the easy versus hard tasks. We also used standard model-fitting indices to gauge statistical fit. The models differed in their complexity (i.e., number of free, or estimated, parameter values). For example, the Biased Model has one free parameter, representing initial predisposition, whereas the Full Model has two additional free parameters, representing task-dependent attentional focus. Therefore, we computed the Akaike Information Criterion (AIC) and Bayesian Information Criterion (BIC) of model fitness, which evaluate model fit while penalizing models that use more free parameters. Thus, the AIC and BIC tend to favor less complex models, all else being equal. Lower AIC and BIC values indicate better fit. To assist with interpretation of our result we also computed AIC weights that roughly estimate the probability of each model being a reasonable account of the data, according to the observed data [79].

When fitting each model, we assumed a binomial error process to connect the predicted choice probabilities to the observed choices. Given the limited number of observations per individual, rather than create a different set of models and model parameters for each individual, we estimated a single set of parameters for each model and used these calculate predictions for all participants. This approach essentially creates a single version for each of the three models tested, which assumes that all individuals shared approximately the same basic decision-making process. In this sense, the models address questions about the typical or “average” decision-maker. However, our analysis did respect individual differences in monetary payoffs earned during each task and individuals’ subjective perceptions of PJSD. That is, to calculate the likelihood that a particular individual would choose the Choice Manager, our models used that individual’s mean payoffs and PJSD ratings within the easier and harder tasks to make its predictions. We used a grid search method to find parameter values that maximized the likelihood of responses according to each model. MATLAB code for this modeling can be found on OSF: https://osf.io/sgpd4/?64405714ed6840a9874f9ef79b0b87c5.

#### Model results

The model results and estimated parameters are shown in Tables 5 and 6. As anticipated, the Full Model provided the best account of the data, with 85% of predicted choices favoring Choice in the easy task (compared to the observed 84%) and 43% favoring Choice in the hard task (compared to the observed 43%). The model indicates that individuals exhibited an initial bias for the Choice Manager. According to this model, the optimal value for this initial preference state, *P*(0), is *ϐ* = 1.00. This value means that participants required substantially less evidence to select the Choice Manager than the No-Choice Manager. Specifically, according to the model, three-times more evidence was required to choose the No-Choice manager. To calculate this difference in evidence, we compared the initial preference state, *P*(0) = 1.00, to the decision threshold, *ϑ* = 2.00. The Choice manager is chosen when *P*(t) ≥ 2, which is 1 point above the initial starting point of 1.00. In contrast, the No-Choice manager is chosen when *P*(t) ≤ -2, which is 3 points below the initial starting point.

**Table 5:** Model fit and predictions.

Model	Log-Likelihood	AIC	BIC	AIC Weight	Observed Preference for Choice: Easier Harder 84%, 43%
					Predictions
1) Null	-113.96	229.96	232.97	0.00	0.60, 0.12
2) Biased	-79.78	163.65	169.64	0.35	0.86, 0.34
3) Full	-77.06	162.38	174.26	0.65	0.85, 0.43

**Table 6:** Estimated parameter values.

Model	*ϑ*	*ϐ*	*w*	*w*_easy_	*w*_hard_
1) Null	2.00	-	-	-	-
2) Biased	3.00	2.00	-	-	-
3) Full	2.00	1.00	-	0.70	0.85

*ϑ* = decision threshold. *ϐ* = bias for Choice. *w*_easy_, *w*_hard_ = potential losses-dependent attentional weighting of outcome utility/procedural utility, for the easy versus hard task.

The Full model also indicated that attention to procedural utility differed across tasks. In the easy task, where most participants earned net gains under the Choice Manager, they were estimated to focus on monetary payoffs (outcome utility) 70% of the time, and freedom of choice (procedural utility) 30% of the time (*w*_*easy*_ = 0.70). In the hard task, where individuals typically earned net losses with the Choice Manager, attention to freedom of choice was reduced by half, with an estimated 85% attention to outcome utility, and 15% procedural utility (*w*_*hard*_ = 0.85).

The Null model, which entirely ignored procedural utility, was the worst performing model, significantly underestimating preference for Choice. This model predicted that 60% of individuals would choose the Choice Manager in the easy task (vs. 84% actual), and only 12% would choose the Choice Manager in the hard task (vs. 43% actual). The Biased Model improved predictive accuracy for the easy task, with 86% of individuals predicted to choose the Choice Manager (vs. 84% actual), but underestimated preference for Choice in the hard task by 9% (34% predicted vs. 43% actual).

Overall, these findings support our hypothesis, and the plausibility of DeCaro et al.’s (2020) [13] original assumption, that attention is drawn to the economic outcome utility dimension when individuals experience losses, causing them to deemphasize freedom of choice (procedural utility). These findings also affirm that individuals incorporate both outcome utility and procedural utility in their decision calculus when deciding preference for Choice. According to our best-fitting, procedural utility model, individuals were initially predisposed to favor the Choice Manager, presumably due to a fundamental preference for choice. However, as individuals began to experience pronounced financial losses, and economic insecurity, their attention shifted to the economics of the situation (i.e., outcome utility), causing many individuals to abandon the procedural utility of Choice for the economic benefit and protection of the No-Choice Manager. The standard economic model, which lacked elements to account for these aspects of behavior, performed poorly, underestimating preference for choice. This was especially true in the hard task, where Choice was associated with financial losses. The standard model assumes that everyone who earns losses in the hard task will abandon Choice. Most individuals did abandon choice in the hard task, as previously noted, but some continued to prefer Choice anyway. This effect was not captured by the standard model.

## Discussion

It is generally thought that individuals prefer freedom of choice (procedural utility) [3,5,35]. However, by virtue of living in societies, humans routinely trade individual liberty (e.g., freedom of choice) for collective safety, economic security, and other benefits provided by parents, employers, educators, governments and other, more efficacious actors [12,15,23,24,27,59]. The decision processes involved in such Faustian bargains are poorly understood [13]. The dominant perspective in decision science—economic rational choice theory—ignores the involvement of procedural utility in human decision-making, assuming that the utility of final, economic outcomes is crucial [8]. These perspectives must be reconciled to properly account for human behavior within the myriad Faustian arrangements that constitute society [13,18,22,80].

We addressed this gap in the current study, examining decision-makers’ preference for choice in a decision task with performance-contingent financial payoffs, spanning well-defined losses and gains. Participants completed both an easy version of the task and a hard version designed to generate failure (i.e., losses), decrease their sense of self-efficacy, and threaten their sense of security. They were supervised by two managers: a Choice Manager that granted personal decision control, and a coercive No-Choice Manager who denied personal decision control but ensured successful performance (i.e., gains) on the hard task. We used mediational analyses and computational cognitive models to better understand the psychosocial (e.g., motivational) and cognitive (e.g., attentional) mechanisms involved in participants’ decision to retain choice or abandon choice in exchange for better financial outcomes.

Participants reported greater procedural utility (i.e., procedural justice and self-determination) when working with the Choice Manager. Participants also felt more satisfied earning gain outcomes with Choice, versus No-Choice, indicating a bonus to felt utility. However, there was no such enhancement for loss outcomes, indicating a weaker procedural utility effect for losses, which fell below the financial goal point ($5). These findings replicate and extend DeCaro at al.’s (2020) [13] original experiment with a new sample, modified decision task involving easier versus harder activities, and a modified preference task, where individuals indicate preference for a Choice versus No-Choice Manager for the easy versus hard task. This study again demonstrates the value of choice above and beyond the financial outcomes earned, while identifying an important, but previously overlooked, boundary condition—losses.

This study also extended DeCaro et al.’s (2020) [13] prior experiment by targeting and assessing efficacy and security, two important factors thought to underpin decision-makers’ decisions to relinquish control to others [12,25]. Most participants performed well when exercising decision control with the Choice Manager in the easy task, quickly learning the optimal response and earning approximately $6.13 (gain), compared to the No-Choice Manager’s pre-determined $5.71 (gain). Importantly, participants reported feeling more efficacious and secure making decisions themselves in the easy task. As expected, most participants (85%) preferred the Choice Manager in this version of the task.

However, these same participants performed poorly in the hard task, failing to learn the optimal response and earning just $3.84 (a loss) under the Choice Manager. The No-Choice Manager continued to perform moderately well in the hard task, earning $5.71 (gain). Importantly, participants reported feeling inefficacious and less secure when making decisions themselves in the hard task. Most participants (57%) preferred the No-Choice Manager, reversing their preference. This reversal was mediated by perceived security.

These findings indicate that participants would rather relinquish freedom of choice to a controlling leader who could protect their security and ensure better outcomes than retain decision control and incur a financial loss. This finding supports prior theoretical speculation that security is a determining factor behind individuals’ decisions to not only grant decision control to others [12,14], but also specifically to coercive others [25,28,29]. This demonstration is important because prior research has been largely non-experimental [e.g., 36,38,39,44]. Furthermore, the few prior experiments have either focused on benign no-choice conditions that lack an authority figure [e.g., 10] or benevolent authorities [45,53]—not coercive authorities who actively seek to limit personal choice.

This research integrates and extends decades of prior research on desire for control, autonomy, procedural justice and economic rational choice [see 4,5,8–15 for review]. To better understand the decision calculus involved in these decisions, we created and compared three computational cognitive models, using Decision Field Theory’s modeling framework [32]. The best, and most accurate, model was a procedural-utility model (i.e., Full Model) that incorporated utility derived from both choice and financial outcomes to determine participants’ preferences when facing various economic prospects. The Full Model included a parameter representing initial preference (predisposition) for choice, and two attentional weights, representing relative attention to procedural utility versus outcome utility, as a function of economic threat (i.e., losses, in the hard task). Overall, participants were estimated to exhibit a strong initial bias in favor of choice, requiring 3-times more evidence to abandon choice. They were estimated to have focused on procedural utility 30% of the time, in the easy task, versus just 15% in the hard task. Increased attention to the economic outcome dimension accounted for participants’ preference reversal, preferring the Choice Manager when the task was manageable (easy), but preferring the No-Choice Manager when the task was too difficult (hard). This model is consistent with the premise that individuals generally prefer choice, but may sacrifice decision control to more efficacious others in challenging situations [e.g., 12,14]. This model is also consistent with substantial research indicating that the threat of loss (e.g., loss aversion, negativity bias), elicits a strong reaction from individuals [33,55], capturing their attention [34], and motivating them to take risks or make sacrifices to mitigate those losses [51,52,57].

The Full model’s predictive accuracy differed substantially from the Null Model, representing standard outcome-based utility. The Null Model must predict preference for whichever manager was associated with higher net earnings. Thus, the Null Model missed observed preference for Choice by 24% in the easy task and 31% in the hard task, where some individuals retained preference for Choice, despite earning less than the No-Choice Manager.

In summary, the current results support the hypothesis that individuals (a) consider both procedural and outcome utility when striking Faustian Bargains, and (b) calibrate their preference for choice contextually, based on initial bias (i.e., default preferences for decision control), anticipated outcomes of decision control (i.e., losses versus gains), performance-contingent needs (e.g., economic security), and their capacity to satisfy those needs themselves (e.g., efficacy). The current study advances prior research in several ways. We discuss these advancements, paying special attention to theory development and future research directions.

### Preference for choice

Research indicates that individuals desire choice [3,5,15] and benefit from fair, autonomy-supportive leaders and institutional decision processes [8,41]. However, research also indicates that there are situations where individuals do not desire or benefit from choice [12,14]. For decades, these perspectives have been treated as antagonistic, representing irreconcilable theoretical stances[4,e.g., 14]. Sufficient evidence exists to firmly conclude that preference for choice is nuanced—depending on decision-maker and context. We, therefore, urge decision scientists to ask *how* decision-makers value choice, *when* they trade choice, and *why* they do so, rather than *if* they value choice.

Many individuals indeed value choice, but there are important factors to consider. First, individuals may be more likely to relinquish decision control when the decision is, for example, among tragic alternatives, uninteresting, unimportant, or too taxing (e.g., too many choices), or the decision-maker feels too inefficacious to handle the decision properly [12,23,45]. Second, there are important individual and cultural differences to consider. These differences arise in the expression and satisfaction of self-determination—different forms of decisional control are perceived and satisfy fundamental needs differently depending on the perceiver and the situation [2,77,81–83]. Finally, the domain in which the Faustian Bargain occurs (e.g., work, government, politics) likely matters, because choice may hold greater or lesser value, and different economic consequences, in different domains. We discuss each of these possibilities next.

#### Domain

Using DFT, we identified a potentially important, basic cognitive model of the Faustian bargain. We chose DFT for two reasons. First, unlike many other utility-based computational frameworks of decision-making, DFT explicitly models the attention mechanism thought to be involved in utility updating during preference formation [32,73]. This feature is ideal for the current study because DeCaro et al. (2020) [13] hypothesized that a valence (e.g., loss) dependent attention (“weighting”) mechanism is central to outcome/procedural utility tradeoffs [cf. 34,55]. Second, DFT has been successfully applied to a variety of decision-making behaviors, under different constraints: preference reversals under time pressure [84], choice paradoxes [85], pricing [73], planning in dynamic decision-making [72,74], multi-attribute choice (involving options with multiple dimensions) [86], and multi-alternative (multi-option) context effects [75]. Thus, DFT provides a flexible framework with which to test current and future hypotheses about the cognitive processes involved in preference for choice, and procedural/outcome utility tradeoffs, in different contexts. The precise form of the Faustian Bargain likely depends on many factors. The effects of these factors could be examined within DFT by observing the effects on DFT’s bias parameter (*ϐ*), attention weight(s) (*w*), and/or decision threshold (*ϑ*).

For example, our results and cognitive model align with observations made in the work sector. Even though individuals value organizational and procedural fairness, and often desire self-determination in the workplace [44,87], most workers are not self-employed. Many people “settle” for adequate-paying jobs, without ideal levels of self-determination or procedural justice [88]. This outlook may differ in other domains. In some contexts (e.g., democracies), individual choice and democratic rights are treated as sacred values [9,89]. Individuals may, therefore, be more reluctant to exchange their freedom of choice for coercive political leaders. In DFT terms, the greater significance assigned to choice (i.e., democratic rights) in the political realm should equate to a greater initial bias (*ϐ*) for choice (i.e., autonomy-supportive, democratic candidates), as well as greater attention (*w*) to procedural utility (i.e., procedural justice, self-determination).

We anticipate that initial bias for Choice (*ϐ*) will be low in domains where individuals are typically decision averse, such as when making tragic decisions (e.g., parents deciding which child must die due to inadequate medical supplies [11]). In addition, because of the dire stakes involved (i.e., life and death), attention (*w*) may be more strongly drawn to outcomes (who lives or dies), rather than procedural fairness and self-determination. More generally, there is a norm for patients to relinquish considerable decision-making authority to doctors, whom many individuals perceive as benevolent experts [43,45]. A similar norm is true in many realms involving experts [12,90]. Hence, individuals likely exhibit different bias for Choice (*ϐ*), decision thresholds (*ϑ*), and/or focus of attention (*w*) in different settings, where they inhabit different roles and, therefore, have different norms and expectations of decisional control [cf. 12,82,91].

#### Culture and individual differences

Preference for Choice is also affected by individual differences and culture. Procedural justice and self-determination are fundamental needs [4,92]. However, there are individual differences in preference for specific forms of choice to satisfy those needs. For example, it is widely believed that collectivistic societies devalue individualistic decision procedures (e.g., direct individual decision control) and prefer collectivistic or relational decision procedures (e.g., deferring to trusted elders, senior authority figures), by default [2,77,82,cf. 83]. This cultural difference in preference for specific types of Choice could be examined experimentally via selective recruitment. We anticipate such cultural differences to manifest as group differences in bias (*ϐ*) for particular kinds of Choice [e.g., 83,93,94]. Race, class, and gender are important factors affecting preference for Choice [82]. For example, following the 9/11 terrorist attack on the U.S. World Trade Center, research indicated that African Americans—a group of individuals who have historically experienced racially-motivated political disenfranchisement—were especially unwilling to sacrifice their civil liberty for national security [36]. We did not see these factors (i.e., gender, race) emerge as significant in the current study. However, we do wish to note descriptively that compared to males, approximately 10% fewer females were willing to sacrifice decision control to the coercive No-Choice Manager in the hard task (female: 46.63% preferred Choice vs. male: 35.00%). Furthermore, compared to individuals who identified as Caucasian, approximately 10% fewer African Americans were willing to sacrifice decision control to the No-Choice Manager (Caucasian: 39.58% preferred Choice vs. African American: 50.00%). Attentional focus (*w*), may also be affected, if there are context-specific (sub)cultural differences in attention to decision processes versus outcomes. The current study was not designed to examine the potential effects of race, class, or gender, nor did this study have sufficient sample size to do so. However, understanding the influence of such factors in future studies will better inform public policy and facilitate better match of governmental systems (especially participatory decision-making processes) to stakeholder preferences [81,82].

### Institutional choice, cooperation, and compliance

The questions we have raised in this study are important to society, because preference for choice—and by extension, particular types of rule-systems (i.e., institutions) that affect choice—influence acceptance, cooperation and compliance [41,89]. These outcomes, in turn, influence provisioning of public goods (e.g., economies, governments, resources) necessary for civilization and human welfare [21,22,95]. For example, decisions about employment and national security, and current societal debates in the U.S. about abortion rights, COVID-19 safety regulations, and government and market systems, are fundamentally debates about tradeoffs between individual rights (e.g., self-determination) versus governmental authority [cf. 8,25,44,80,96,97]. The decision outcomes of these debates strongly influence subsequent acceptance, compliance, and cooperation.

For example, to understand how governmental autonomy-support affected compliance motivation during the first wave of the U.S. 2020 COVID-19 pandemic, DeCaro and DeCaro (2022) [98] surveyed approximately 800 individuals (400 Republicans, 400 Democrats) in California, Florida, Texas, and New York. They found that most individuals perceived government leaders (e.g., state governors and their administration) from opposing political parties as coercive (i.e., low in procedural justice and self-determination). This perception correlated with decreased self-reported motivation and compliance with voluntary (advisory) and mandatory (enforced) safety guidelines (e.g., state-at-home orders). However, some of these individuals did perceive opposing political leaders as autonomy-supportive. These individuals exhibited greater motivation and compliance, as well as security—especially if safety guidelines were enforced. This effect was strongest for Republicans, who otherwise did not prefer mandatory guidelines.

Acceptance, motivation, and compliance appeared to be heightened in the governmental situation that provided the most procedural utility [cf. 6,8]. Preferences for different forms of government are likely to be some function of perceived procedural utility and security [cf. 8,17–18,82,92]. DFT could be useful in understanding these preferences. For example, it has been widely argued that preferences for coercive governance systems rise in moments of extreme crisis [cf. 24–28,25–40]: decision-makers may shift attention, *w*, to the security dimension (emphasizing safety or economic survivability), deemphasizing the procedural justice/self-determination dimension. Cultural or individual preferences for particular forms of government (emphasizing different tradeoffs among individual liberty and security) may likewise be captured by initial bias, *ϐ*. Hence, the conceptual and methodological innovations introduced in the current study may offer novel ways to examine classical and important questions about institutional preferences.

### Limitations

We assumed that attentional focus was an important feature in determining the influence of procedural versus outcome utility. However, we did not measure direction and level of attention. Future studies could provide greater insight into these covert attentional processes using eye-tracking and other process-tracing techniques [34,99,100]. Advances in the joint modeling of behavioral and neural data [e.g., 10,101] may also provide novel avenues for elucidating the cognitive processes involved. Finally, we do not present the current cognitive model as canonical or definitive. It is possible to envision many different cognitive models to describe any dataset. We sought to test three of the most obvious models, keeping within the constraints of our study design and data. However, as we have pointed out, numerous factors may affect the importance or involvement of particular parameters within the model. We encourage future researchers to explore these possibilities.

### Conclusion

As many prominent scholars, politicians, and scientists have stated, human decision-makers inherently face a fundamental paradox: choice is valuable [61] but not without cost [14]. This paradox—a Faustian bargain [18]—manifests in human cognition as an inescapable decision: where to strike the balance between individual liberty and security? More research is needed to systemically address this question. Decision science must be firmly based on the recognition that “choice” (i.e., exercising choice) typically occurs in a “choice context,” forcing decision-makers to reconcile their need for self-determination with economic security and survival.

## Supporting information

S1 File (DOCX)

S1 Dataset (XLSX)
