# Supplementary material for: Formalizing the fundamental Faustian bargain: Inefficacious decision-makers sacrifice their freedom of choice to coercive leaders for economic security
Source: PLoS One. 2022 Sep 27;17(9):e0275265. doi: 10.1371/journal.pone.0275265 (PMC9514627; doi:10.1371/journal.pone.0275265)
Supplement: S1 File — (DOCX) [file pone.0275265.s001.docx]

**SUPPORTING INFORMATION**

**Formalizing the fundamental Faustian bargain: inefficacious decision-makers sacrifice their freedom of choice to coercive leaders for economic security**

Table of Contents

[S1.0 STUDY MATERIALS 2](#_Toc108613898)

S[1.1 Task Instructions 2](#_Toc108613899)

S[1.1.1 Part 1: Easy Task 2](#_Toc108613900)

S[1.1.2 Part 2: Hard Task 10](#_Toc108613901)

S[1.2 Technical Design Elements 12](#_Toc108613902)

S[1.2.1 Decision Trials and Payoffs: Easy Task 12](#_Toc108613903)

S[1.2.2 Decision Trials and Payoffs: Hard Task 14](#_Toc108613904)

S[1.2.3 Manager Statements of Autonomy-Support vs. Coercion 17](#_Toc108613905)

S[1.3 Psychological Measures 18](#_Toc108613906)

S[2.0 SUPPLEMENTAL ANALYSES 19](#_Toc108613907)

S[1.1 Order Effects 19](#_Toc108613908)

S[1.2 Outcome Satisfaction 20](#_Toc108613909)

# S1.0 STUDY MATERIALS

In this section, we provide detailed technical description of the experimental design, including task instructions, underlying payoff distributions, and measures (e.g., survey items).

## S1.1 Task Instructions

This section contains the task instructions for both the card-based outcome satisfaction task, which was the main decision-making/performance task, and the choice tradeoff task (i.e., Faustian bargains), in which individuals subsequently indicated their preference for the autonomy-supportive Choice Manager versus unsupportive No-Choice Manager. The experiment was divided into both an easier and harder version of the task, broken down into two phases (labeled Part 1, Part 2). The order of task difficulty (easier, harder) was counterbalanced across participants. Order 1 (Part 1: easier, Part 2: harder) is reported here for illustration. All materials were presented on the computer, using *E-Prime* 2.0.

### **S1.1.1 Part 1: Easy Task**

**Cover Story**

*Said to participant at beginning of experiment, when introducing the study (during Informed Consent)*

Today you will be asked to do a card-based decision task on the computer, and answer a few follow up questions about the task.

You will receive research credits for your psychology course. And, you will also be paid $1 to $9 based on your performance on the card task.

We will start with some instructions on the computer that explain the details of the study, what you’ll be asked to do today, and your payment. It is very important that you read all of the instructions carefully. We will test your understanding of the instructions after you read them.

**Introductory Instructions** [*Screen 1*]

Introductory Instructions

Welcome and thank you for your help today.

Learning to make good decisions is an essential part of life and work. The card task you will be doing today attempts to provide an assessment of your decision making ability.

**Task Overview** [*Screen 2*]

Task Overview

The Card Task you are doing today consists of two parts: Part 1 and Part 2. Your payment today will be based on your performance across both parts of the card task. We will determine your average score across all trials of the card task, and this average will be your final payment.

We are also interested in your perceptions of different kinds of decision situations. The decisions you make today will be followed by some brief questions.

Please try your best on the Card-based Decision Task, and respond as honestly as possible to any follow-up questions you receive about your perceptions of the task.

We will explain Part 1 of the Card Task now, and explain Part 2 later.

**Card Task Part 1** [*Screen 3*]

CARD TASK: PART 1

Decks

The cards in each deck below have been assigned a monetary value ranging from $1 to $9. **Each of the 4 decks has its own average payout**, so some decks have higher paying cards on average than other decks.


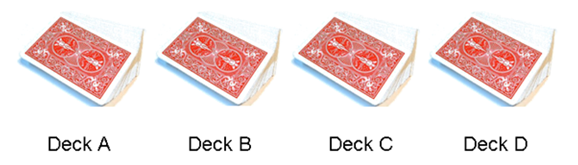


**Decision Task** [*Screen 4*]

Decision Task

The Card Task measures your decision-making performance based on your ability to get good payoffs. Your task is to learn enough about the decks’ payoffs to choose a deck accordingly.

Your score will be determined by the typical payoff you earn across the entire task (i.e., your AVERAGE payoff). This will indicate whether you, on average, made good decisions. You can increase or decrease your score depending on your ability to learn to make better decisions.


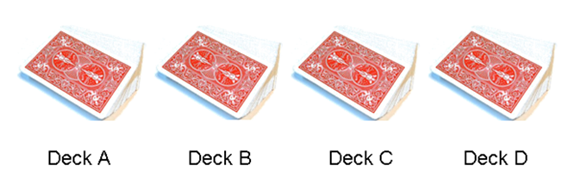


**Controls** [*Screen 5*]

How to enter your choice on each trial

This task includes guidance during each decision you make. The guidance is in the form of a computerized manager. Begin each decision by reading the advice given here first.


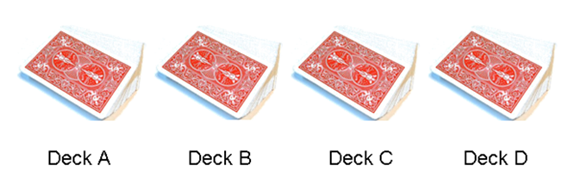


To select a deck during the task, press the corresponding key on the keyboard. The keys are labeled (A, B, C, and D).

**Performance** [*Screen 6*]

Performance Standards

We have established certain performance standards in this task. These standards are explained next.

Note that today we will be keeping track of your performance.

**Performance Continued** [*Screen 7*]

Performance Standards

$5 Typical Performance [bolded]

The majority of people score $5. Individuals that score $5 are considered to have standard decision- making performance.

$4, $3, $2, or $1: Scoring further and further below typical performance indicates poorer and poorer decision making.

$6, $7, $8, or $9: Scoring further and further above typical performance indicates better and better decision making.

**Summary** [*Screen 8*]

Summary of Performance Standards

Please review these evaluation standards carefully. Once you have committed them to memory, please continue.


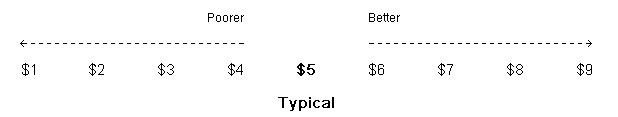


Because you will make many decisions during this card task, the best way to keep track of your performance is based on the outcome of each INDIVIDUAL decision trial. For example, if you consistently score $5 across trials you will likely score an average of $5 (Typical score).

**Payment** [*Screen 9*]

Your Payment

Many real-world decisions have financial outcomes or consequences.

Thus, to make the simulation of decision settings as realistic as possible, you will earn money today based on your decision-making performance.

You will receive this payment in addition to any research credits you earn by participating in this study.

Your payment will be explained next.

**Payment Continued** [*Screen 10*]

We will pay you based on your performance.

Your score today is the AVERAGE of all the payoffs you receive during the card-based decision task. Your payment today will equal this score.

Examples:

- If you score an average of $1, we will pay you $1.
- If you score an average of $5, we will pay you $5.
- If you score an average of $9, we will pay you $9.

The best way to keep track of your performance is based on the monetary payoff you receive after each INDIVIDUAL decision trial. For example, if you consistently receive $5 across trials you'll be paid $5.

**Instruction Quiz** [*Screens 11-15*]

*Participants completed an automated quiz before beginning the card task. Questions were presented on separate screens. The computer provided automatic feedback, with correct responses.*

PERFORMANCE & PAYMENT STANDARDS

Before you get started we want to verify that you understand how your performance on the card-based decision-making task will be evaluated today.

Please type your answer (numerical values only):

1. What score on the decision task is considered *typical of most people*? $_______
2. What will we pay you if you score an average of $5 on the Card Task? $_______
3. What will we pay you if you score an average of $1 on the Card Task? $_______
4. What will we pay you if you score an average of $9 on the Card Task? $_______

**About to Start Part 1** [*Screen 16*]

You will begin Part 1 of the actual decision task on the next screen!

IMPORTANT!!

From this point forward we ask that you do your best to achieve BOTH (1) a strong decision making score and (2) high personal monetary earnings.

Please open the door and wait for the experimenter to start the program.

**Experimenter: After Quiz**

*Immediately after the quiz, participants were instructed to get the experimenter. The experimenter then answered any questions they had and summarized some important task details.*

[*Questions?*]

Do you have any questions about the decision task?

[*Payment Receipt*]

Now we’ll fill out the receipt for your payment today. Please get the Payment Form that is on top of the computer tower. Write your contact information, but leave the payment amount blank until the end of the study. After you complete the decision task, I will see your score and pay you based on that score. You can leave the completed payment form on the desk until then.

[*Review Important Instructions*]

I’ll go over a few points with you before we begin the Card Task.

The number keys on your keyboard have been labeled to allow you to enter the numbers 1 through 11. Also the keys labeled A, B, C, and D correspond to the 4 decks in the decision task.

You can begin when you’re ready. [*Experimenter enters code to activate Part 1*]

If you have any questions at any point, just let me know.

*[Experimenter left the room. Waited in the waiting area*.]

**Example Trial: Card Task (Decision)**

*Trial with autonomy-supportive Choice Manager shown.*


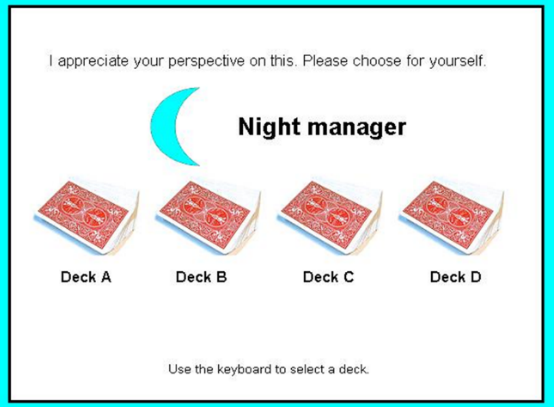


**Example Trial: Card Task (Monetary Payoff and Outcome Satisfaction Rating)**

*Payoff and outcome satisfaction rating screen appeared immediately after a decision trial.*


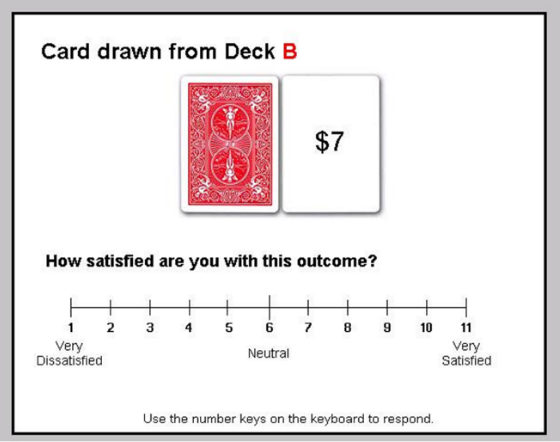


**Follow-up Questions Instruction Screen**

*After the final trial of the card task, participants completed the choice tradeoff task and responded to the follow-up questions.*

Follow-up Questions

Before we move on to the next part of the experiment, we would like to ask you a few questions about Part 1 of Card Task that you just completed.

Your responses to these questions will NOT change your scores (or payment). We greatly appreciate your genuine feedback, so please be as honest as possible in reporting your reactions.

**Preference Task**


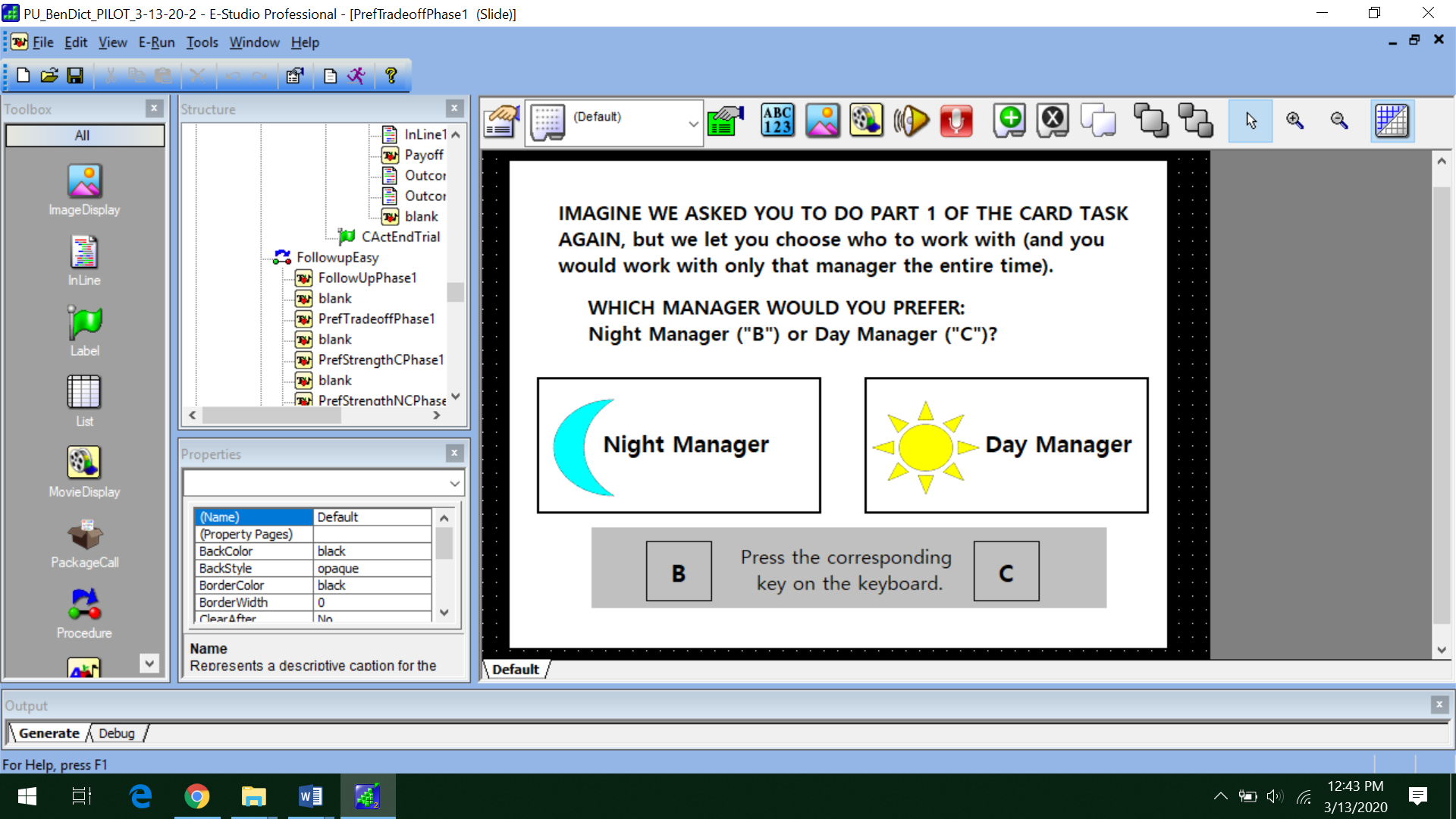


**Preference Strength**

*Preference strength items (one for each manager) were shown on separate screens. Night Manager is shown as the example.*


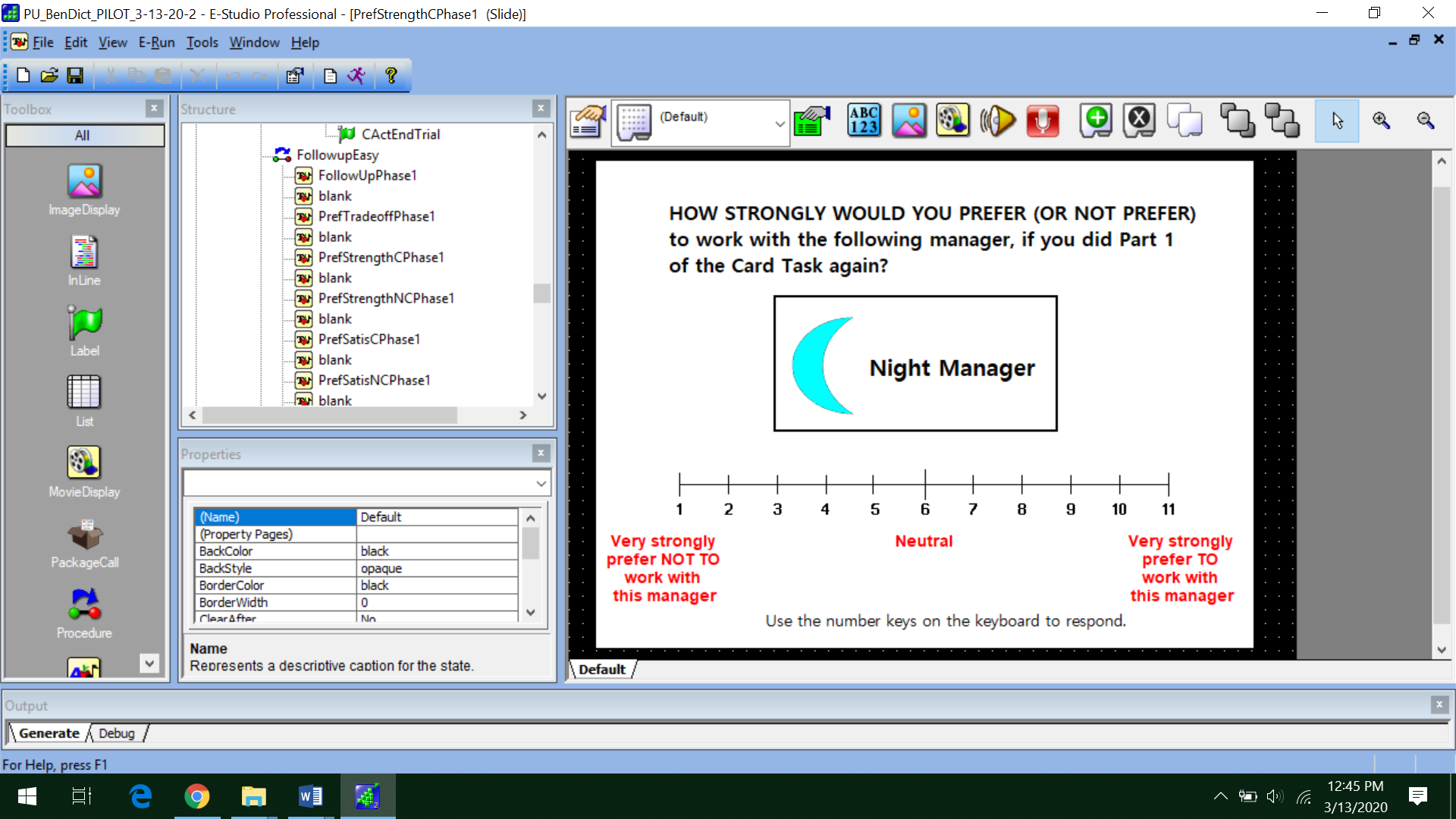


**Manager Satisfaction**

*Anticipated satisfaction working with each manager was assessed on separate screens. Night Manager is shown as the example.*


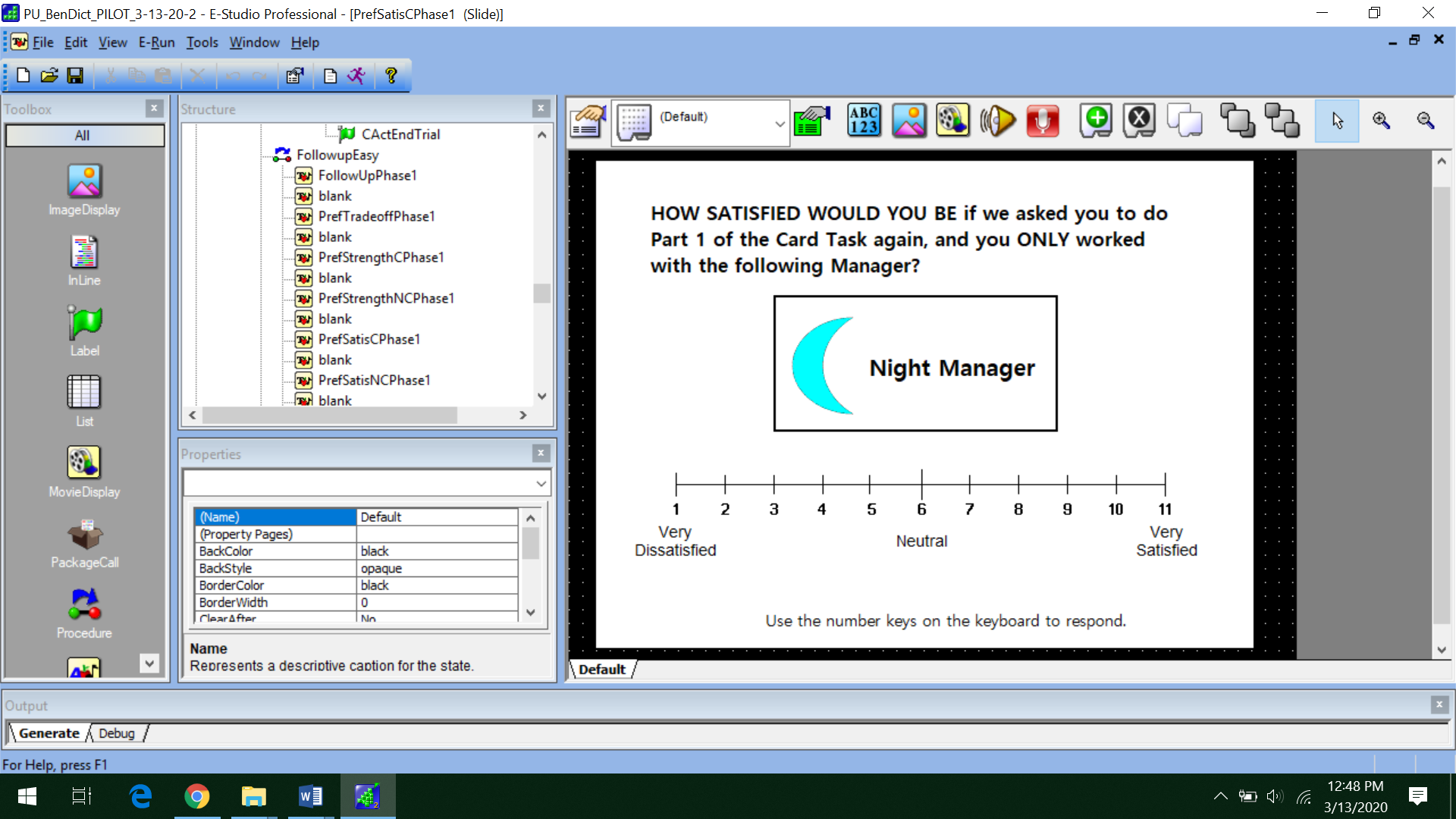


**Correct Response**

*We asked participants to indicate the correct/optimal response for the card task they just completed.*


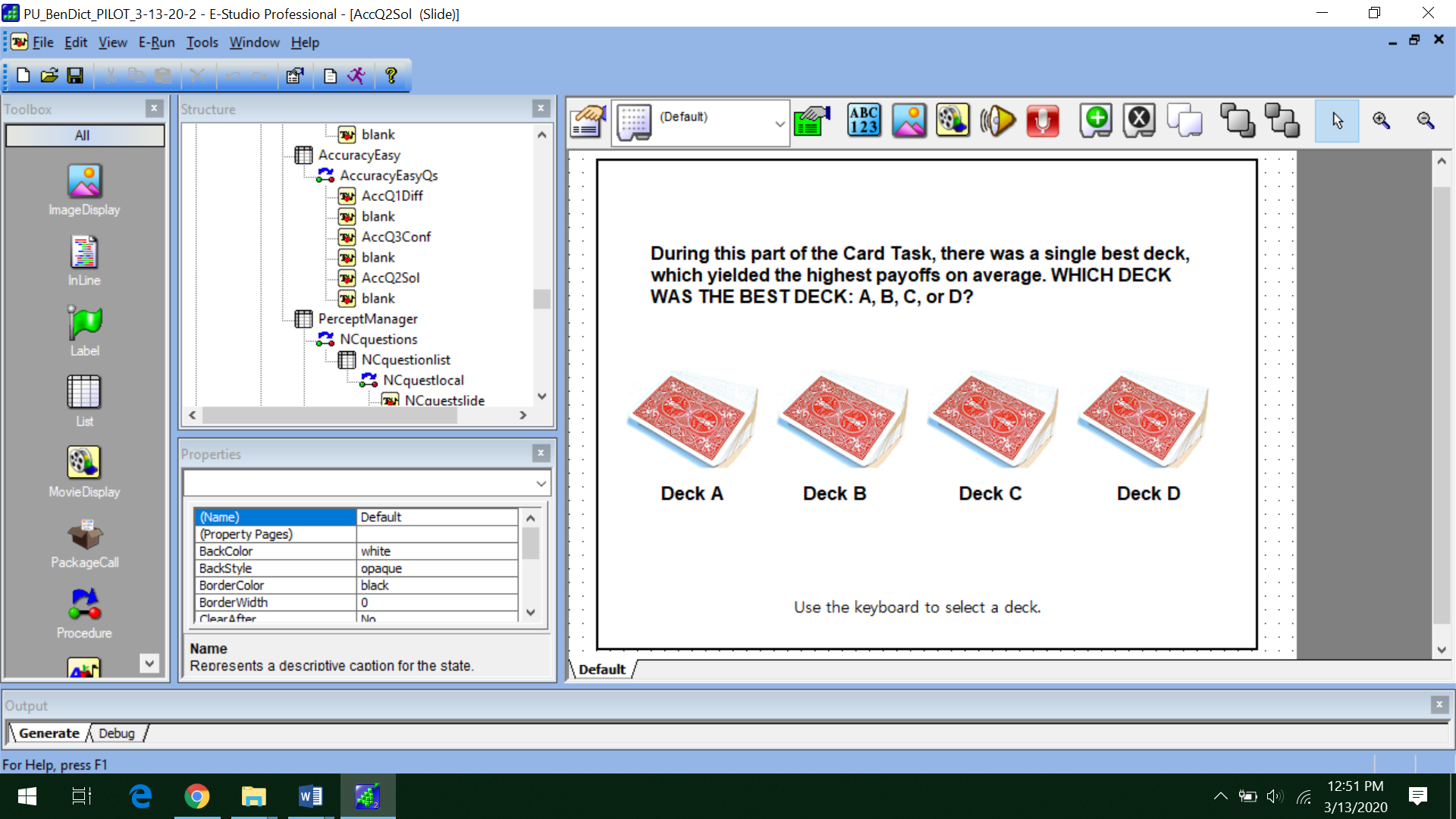


**Psychological Measures Screen**

*Follow-up questions assessing psychological reactions to the card task (e.g., self-determination, security) were assessed using the following screen template. The computer automatically presented each item individually in the [questions] field (selected at random from the underlying question list). Example shows the Night Manager.*


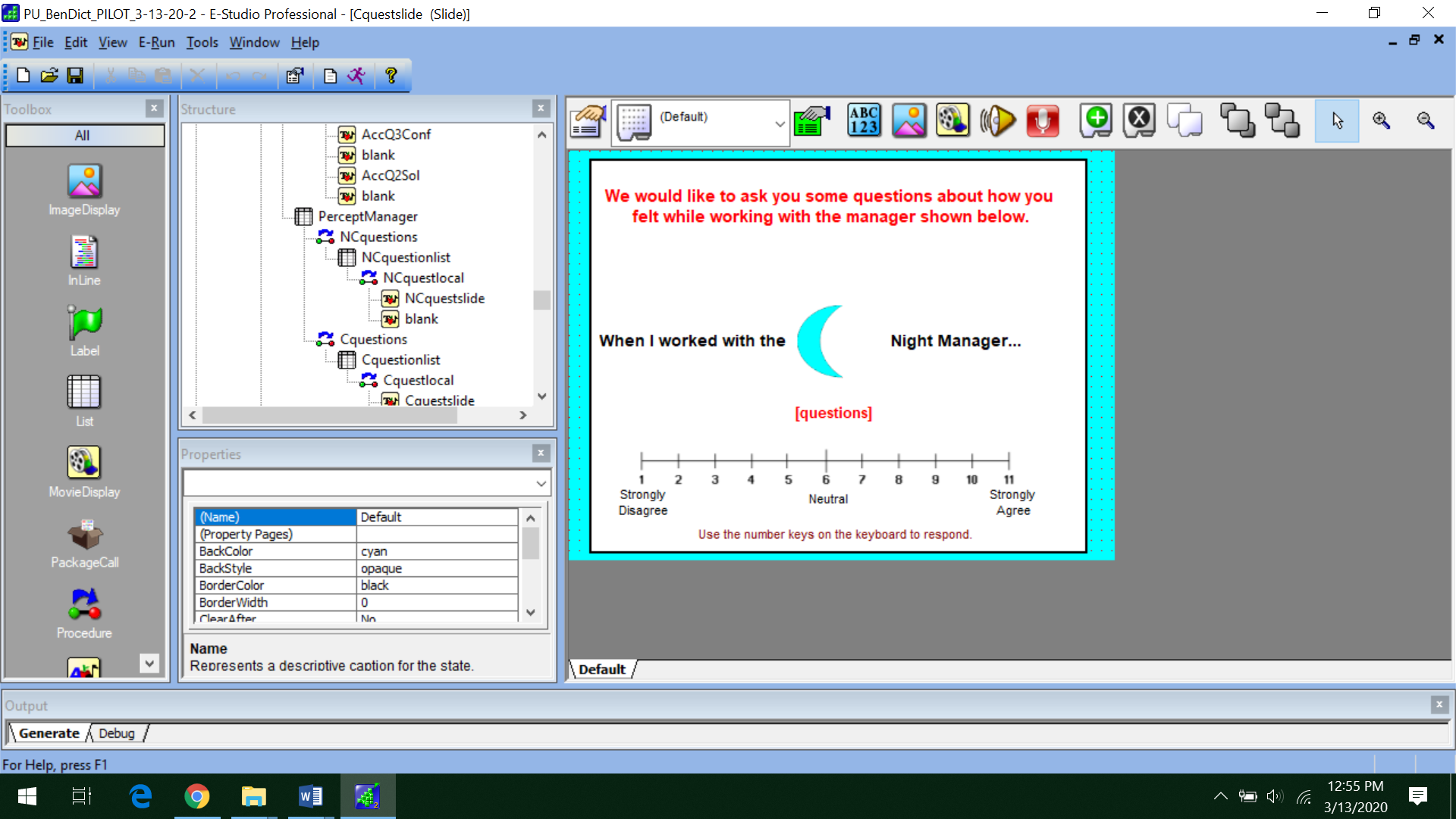


### **S1.1.2 Part 2: Hard Task**

**Introducing Part 2** [*Screen 1*]

*After completing all follow-up questions to Part 1, the computer allowed a rest period. When ready, participants continued to Part 2.*

Task Overview

Next, we will do Part 2 of the Card Task.

**Card Task Part 2** [*Screen 2*]

CARD TASK: PART 2

Sequence

In this part of the Card Task**, there is a pattern or sequence** for getting the best outcomes. In other words, you must select decks in a particular order. Your goal is to learn the pattern.

Each time you select the correct deck in the sequence you will typically receive one of the higher payoffs ($6 to $9).

Each time you select the wrong deck in the sequence you will typically receive one of the lower payoffs ($1 to $4).


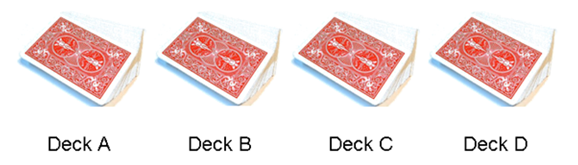


**Decision Task** [*Screen 3*]

Decision Task

Like before, this part of the Card Task measures your decision-making performance based on your ability to get good payoffs. Your task is to learn the pattern well enough to choose accordingly. You can increase or decrease your score depending on your ability to learn to make better decisions.


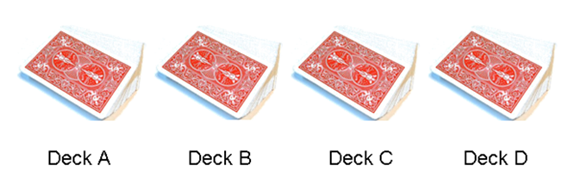


**Controls** [*Screen 4*]

Managers

**You will receive guidance from the same two managers you had before.** Begin each decision by reading the advice given here (above the decks) first.


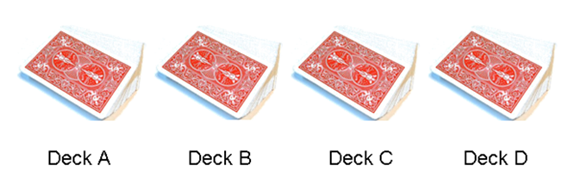


To select a deck, press the corresponding key on the keyboard.

**Performance** [*Screen 5*]

**The performance standards for Part 2 are as the same as before:**

$5 is Typical Performance: A score of $5 is considered standard decision-making performance.

$4, $3, $2, or $1: Scoring further and further below typical performance indicates poorer and poorer decision making.

$6, $7, $8, or $9: Scoring further and further above typical performance indicates better and better decision making.


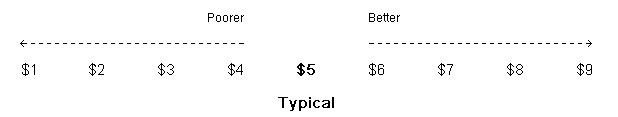


The best way to keep track of your performance is based on the outcome of each INDIVIDUAL decision trial. If you consistently score $5 across trials you will likely score an average of $5 (Typical score).

**Payment** [*Screen 6*]

A Reminder About Your Payment

Your score today is the AVERAGE of all the payoffs you receive during the card-based decision task. Your payment today will equal this score.

- If you score an average of $1, we will pay you $1.
- If you score an average of $5, we will pay you $5.
- If you score an average of $9, we will pay you $9.

The best way to keep track of your performance is based on the monetary payoff you receive after each INDIVIDUAL decision trial. If you consistently receive $5 across trials you'll be paid $5.

**About to Start Part 2** [*Screen 7*]

You will begin Part 2 of the actual decision task on the next screen!

IMPORTANT!!

From this point forward we ask that you do your best to achieve BOTH (1) a strong decision making score and (2) high personal monetary earnings.

Please open the door and wait for the experimenter to start the program.

**[*All subsequent screens were the same as Part 1.*]**

## S1.2 Technical Design Elements

In this section we describe technical details of the design, including underlying payoff distributions and manipulations (outcome valence: losses, gains; decision control: choice, no-choice).

### **S1.2.1 Decision Trials and Payoffs: Easier Task**

**OPTIMAL RESPONSE:**

During the easier version of the card task, participants must try to learn and choose the single deck with the highest average payoffs. The best deck is Deck D, with an Expected Value (EV) of $8.70 (high gains). Deck B’s EV is $5.00 (status quo), and Decks A and C are both losses decks with an EV of $2.00.

Each deck is designed to have some variability in the individual payoffs, simulating a probabilistic system (see Payoffs described further below).

**MANAGER PERFORMANCE:**

In this experiment, the No Choice Manager is designed to perform moderately well on the task, so that most participants can perform better than the No-Choice Manager during the easier task, but worse than that manager during the harder task.

**Participant’s Decisions (Choice Trials):**

During Part 1, there will be a total of 81 trials per condition (choice, no-choice) for a total of 162 trials. This is to ensure sufficient learning of the decks.

**No-Choice Manager’s Decisions (No-Choice Trials):**

The No-Choice Manager is designed to be moderately accurate, drawing from the best deck (Deck D: $8.70) 56% of the time and the second-best deck (Deck B: $5), representing the status quo, 22% of the time, for an overall accuracy of 78% (specifically 77.78%).

**Table S1. Overview of No-Choice Manager’s performance (easy task)**

|  | |  |  |  |  |
| --- | --- | --- | --- | --- | --- |
|  | Deck A | Deck B | Deck C | **Deck D** | *Totals:* |
| Average $: | $2.00 | $5.00 | $2.00 | $8.70 |  |
| Percent Choosing: | 11.11% | 22.22% | 11.11% | 55.56% | 100% |
| Trial Count: | 9 | 18 | 9 | 45 | 81 |

**PAYOFF DISTRIBUTIONS:**

**Diagnostic Trials (Signal versus Noise):**

During Choice trials, participants could potentially concentrate on a particular deck and sample a more limited range of outcomes. We therefore ensured that participants received a minimum set of common outcomes across Choice and No-Choice trails for comparability.

Fifty-four trials (33%) are designed so that each value $1:$9 is presented 3 times in each condition (choice, no-choice), regardless of the deck chosen. These values were used to compare outcome satisfaction levels across conditions. Outcomes from the other trials (108 trials, 67%) came directly from the chosen deck’s underlying payoff distribution (see *No-Choice Payoffs* for example). By including the diagnostic trials, we essentially added additional variability (noise) to the decks (signal).

With 81 trials per condition, there were 54 signal trials (67%) and 27 noise trials (33%). To ensure that the diagnostic, noise trials are evenly distributed across the task, we divided each Phase (e.g., Part 1) into 3 equal blocks (unknown to participants), and distributed the noise trials equally across them: specifically, the payoffs $1, $2, $3, $4, $5, $6, $7, $8, $9 were shown during each block). These 9 noise trials per block were restricted to exhaust the full set of the values $1:$9 before continuing to the next block. This design feature ensured that each participant received the full set of 9 diagnostic outcomes($1 through $9) in each condition each block.

**No-Choice Manager’s Performance and Payoffs:**

In the No-Choice condition, the manager’s choices were pre-determined (see Table S2): approximately 11% Deck A, 22% Deck B, 11% Deck C, 56% Deck D. To ensure that all participants received the same payoffs on signal trials, and the correct proportion of noise trials distributed as evenly and proportionally across decks as possible, we set a specific number of noise trials for each deck. For example, the dictator chooses Deck D approximately 56% of the time (15 trials) each block: 10 (67%) are signal trials, 5 are noise (33%). When accounting for both noise and signal trials, this results in an E(V) of $2.99 for Deck A and C, $5.00 for Deck B, and $7.08 for Deck D. Overall, this yields an E(V) of $5.71 (small gain).

**Table S2. No-Choice Manager’s decisions and payoffs per block (easy task).**

| **Chooses:** | **Deck A**  (3 trials: 11.1%) | **Deck B**  (6 trials: 22.2%) | **Deck C** (3 trials: 11.1%) | **Deck D**  (15 trials: 55.6%) |
| --- | --- | --- | --- | --- |
| **Signal Trials** | 2 trials (67%) | 4 trials (67%) | 2 trials (67%) | 10 trials (67%) |
|  | Block 1: $2, $3 | Blocks 1, 2, 3: | Block1: $1, $2 | Blocks 1, 2, 3: |
|  | Block 2: $1, $3 | $4, $5, $5, $6 | Block 2: $1, $3 | $7, $7, $7, |
|  | Block 3: $1, $2 |  | Block 3: $2, $3 | $8, $8, $8, |
|  |  |  |  | $9, $9, $9, $9 |
|  | *E(V):* $2.00 | *E(V):* $5.00 | *E(V):* $2.00 | *E(V):* $8.10 |
|  |  |  |  |  |
| **Noise Trials** | 1 trial (33%) | 2 trials (33%) | 1 trial (33%) | 5 trials (33%) |
|  | *Random* ($1:$9) | *Random* ($1:$9) | *Random* ($1:$9) | *Random* ($1:$9) |
|  | *E(V): $5.00* | *E(V): $5.00* | *E(V): $5.00* | *E(V): $5.00* |
| **Final E(V)s:** | Deck A | Deck B | Deck C | Deck D |
|  | *$2.99*  *.67($2) signal .33($5) noise* | *$5.00*  *.67($5) signal .33($5) noise* | *$2.99*  *.67($2) signal .33($5) noise* | *$7.08*  *.67($8.10) signal*  *.33 ($5) noise* |
| *TOTAL:* | $5.71 = .11($2.99) + .22($5.00) + .11($2.99) + .56($7.08) | | | |

*Note.* Trials: 27×2 per block = 54 trials. Total Trials: 54 trials × 3 blocks =162.

**Participant’s Performance and Payoffs (Choice Manager):**

In the Choice condition, participants are free to choose whichever deck(s) they want. Therefore, we cannot anticipate exactly the proportion of trials they choose each deck. However, we can roughly anticipate the bounds of participants’ choices and earnings, based on performance observed in DeCaro et al.’s (2020) prior experiment using the same basic task.

The lower and upper bounds of performance in the Choice Condition are defined by two potential scenarios, a scenario where the participant choose completely at random (i.e., does not learn the deck payoff contingencies), and a scenario where the participant learns perfectly the contingencies, and therefore, that Deck D is the dominant option.

When the participant ***chooses completely at random***, the participant will choose Deck D only 25% of the time (Deck B and D, 50% of time), and noise trials will, therefore, be evenly distributed among all four decks on average (approximately 2 noise trials per deck). Hence, the overall E(V) of the Choice condition will be $4.52 ($1.19 less than the No-Choice Manager’s $5.71). This value ($4.52) also corresponds roughly to the interval of the first loss on the scale, falling just below the $5.00 status quo reference point.

Overall E(V) of Choice *Completely at Random:*

**$4.52** = .25($2.99) + .25($5.00) + .25($2.99) + .25($7.08)

In contrast, if the participant ***always chooses Deck D*** (all trials), then all 9 of the noise trials would occur within Deck D. The overall E(V) of choice would be $7.08 ($1.37 more than the Dictator’s $5.71). This value also falls within the interval of moderately high gains (between $7 and $8).

Overall E(V) Choice *Completely Deck D:*

**$7.08** = 0($2.99) + 0($5.00) + 0($2.99) + 1($7.08)

Thus, the most that participants can possibly earn in the Choice Condition is $7.08, and the least we can *realistically* expect participants to earn is $4.52 on average. Participants could, of course, earn even less if they only chose Deck A or C for all trials; however, that behavior seems very unlikely.

DeCaro et al. (2020) observed that most participants learned the optimal deck by the end of Block 1 (i.e., after approximately one-third of the trials). Thus, we ***realistically expect participants*** to learn this contingency relatively quickly (by the end of Block 1), and then choose only Deck D during Blocks 2 and 3. If this pattern is the case, then the likely E(V) for the Choice condition, for most participants will be approximately $6.22 ($0.51 higher than the No-Choice Manager’s $5.71).

Overall E(V) *Realistic Learning Curve:*

$6.22 = [$4.52 (Block 1) + $7.08 (Block 2) + $7.08 (Block 3)]/3

### **S1.2.2 Decision Trials and Payoffs: Harder Task**

**OPTIMAL RESPONSE:**

During the harder task, participants must learn and execute a complex 9-sequence pattern, drawing from the correct deck on the correct trial. The correct sequence is Decks B, A, C, D, D, A, C, B, A. Participants do not have to get the entire sequence correct to earn good outcomes; they will receive good outcomes for each part of the sequence they get correct. Correct trials will yield outcomes predominantly from the gains pool (mimicking Deck’s B and D in Phase 1). However, it is *not* sufficient to simply enter the correct sequence: each part of the sequence must be entered on the correct trial number (thereby making this task considerably more difficult). Thus, to get the first 3 steps of the sequence correct, Deck B must be chosen on Trial 1, Deck A on Trial 2, and Deck C on Trial 3. Failure to draw from the correct deck at the right time will yield outcomes from the losses pool (mimicking Decks A and C in Phase 1). The underlying payoffs (and E(V)) of these pools match those used in the easier task.

**MANAGER PERFORMANCE:**

The harder version of the card task considerably more difficult than in the easier task. The No-Choice Manager is designed to outperform participants.

**No-Choice Manager’s Decisions (No-Choice Trials):**

The No-Choice Manager is designed to be moderately accurate, executing the correct sequence (i.e., drawing from the correct deck on the correct trial) approximately 78% of the time. To outperform the No-Choice Manager, participants would have to hold a rather large sequence and the trial number in memory as they attempt to learn and execute the correct solution.

To implement this design, we segmented each block of the harder task into 6 runs of nine-trial segments (Table S.3), for a total of 54 trials per block (i.e., 6 runs x 9 trials = 54 trials per block). Thus, like the easier task, there were 162 total trials (81 Choice, 81 No-Choice) divided into 3 blocks. And, during each block (54 trials), there were 27 choice trials and 27 no-choice trials.

The No-Choice Manager (Table S.3) gets 21 out of 27 trials (i.e., 77.78%) correct per block, for a total of 63 correct out of 81 No-Choice trials, for an overall accuracy of 77.80%. The placement of errors within each run was designed to simulate a No-Choice Manager that knows the first 6 steps of the sequence well (approximately 90% accuracy overall), but not the last 3 steps (59% accuracy). On incorrect trials, the No-Choice Manager selected a deck from the pool of incorrect decks at random (with replacement). For example, during Run 1, the No-Choice Manager chooses the wrong deck on Trial 3. The correct deck for Trial 3 is Deck C, so the manager would choose Deck A, B, or D at random. This simulates a random search. These features were intentional to convey that the manager indeed knew most of the pattern, yet still make it difficult for participants to easily learn the pattern from the No-Choice Manager’s decisions. This precaution helped to ensure that participants did not outperform the No-Choice Manager.

**Table S3. No-Choice Manager’s accuracy per block (hard task).**

|  | **Trial** | | | | | | | | |  |
| --- | --- | --- | --- | --- | --- | --- | --- | --- | --- | --- |
| **Run** | 1 | 2 | 3 | 4 | 5 | 6 | 7 | 8 | 9 | **% Correct** |
| 1 | **B** | A | C | D | D | **A** | **C** | B | **A** | 4/5 = 80% |
| 2 | **B** | A | C | **D** | D | A | C | **B** | A | 3/4 = 75% |
| 3 | B | **A** | **C** | D | D | **A** | C | **B** | A | 4/5 = 80% |
| 4 | B | A | **C** | **D** | **D** | A | C | B | A | 3/4 = 75% |
| 5 | **B** | **A** | C | D | D | A | **C** | B | **A** | 4/5 = 80% |
| 6 | B | A | C | D | **D** | **A** | C | **B** | A | 3/4 = 75% |
|  |  |  |  |  |  |  |  |  |  | 21/27=77.78% |

The correct 9-trial entry sequence is decks BACDDACBA. Bolded entries are trials the No-Choice Manager gets correct. Underlined entries are Choice Trials (the participant must try to choose the correct deck for the ongoing sequence).

**Participant’s Decisions (Choice Trials):**

We expected, and confirmed during piloting, that most participants would do poorly on the harder task due to the large size of the sequence (9 entries), randomization of Choice/No-Choice trials, and variability of signal (77%) to noise (23%) in payoffs. If anything, participants may learn to correctly enter the first 3 steps of the sequence (B, A, C; 33.33% accuracy), or possibly one to two more entries, after many trials. It is highly unlikely that participants learn the last 3 steps of the sequence.

**PAYOFF DISTRIBUTIONS:**

Payoff distributions for the harder version of the card task were designed to match those used in the easier task as closely as possible. For the No-Choice Manager, the payoff distributions were exactly matched. For Choice, payoffs are more probabilistic by the very nature of the task but still match E(V)s across participants as a whole.

**No-Choice Manager’s Performance and Payoffs:**

Like Phase 1, the Dictator’s overall earnings in Phase 2 will be $5.71, corresponding to an overall accuracy of approximately 78% (Table S4).

***Noise:*** Overall, there were 162 trials (81 Choice, 81 No-Choice). Therefore, to maintain the same percentage of correct/incorrect trials and signal/noise trials as in the easier task, there had to be a total of 27 noise trials (i.e., 33%), and these had to be distributed proportionally across correct and incorrect trials. There are 63 correct trials (corresponding to 78% accuracy), and 18 incorrect trials. Therefore, there had to be a total of 6 noise trials for the 18 incorrect trials (33%), and 21 noise trials for the 63 correct trials (33%), for an overall average noise of approximately 33% (i.e., 27/81 = 33%).

To match the pacing of payoffs used in the easier task, the noise trials in the harder task must also be distributed as evenly as possible across the first, second, and third blocks of the harder task. Thus, as in the easier task, we ensured there was one complete set of the noise values $1:$9 per block.

***Signal:*** Payoffs for signal trials for the No-Choice Manager exactly match the full set of outcomes in the easier task’s Deck B (status quo) and Deck D (high gains) payoff distributions, except that these payoffs were now linked to correct sequence entries, not particular decks. Thus, during correct trials, payoffs were randomly drawn (without replacement) from the combined status quo/high gains pools, until all such values were exhausted for that block. During incorrect trials, outcomes were randomly drawn (without replacement) from the losses pool (equivalent to Decks A and C in the easier task).

**Table S4. No-Choice Manager’s payoffs per block (hard task)**

|  | **SIGNAL (18 Trials)** | |  |  |  |  |
| --- | --- | --- | --- | --- | --- | --- |
|  | Blocks 1, 2, 3 | |  |  |  |  |
|  | Payoffs: | Count |  |  |  |  |
|  | $9 | 4 |  |  |  |  |
| Correct Trials  (14 trials) | $8 | 3 |  |  |  |  |
|  | $7 | 3 |  |  |  |  |
|  | $6 | 1 |  |  |  |  |
|  | $5 | 2 |  |  |  |  |
|  | $4 | 1 |  |  |  |  |
|  | Block 1 | | Block 2 | | Block 3 | |
|  | Payoffs: | Count | Payoffs: | Count | Payoffs: | Count |
| Incorrect Trials  (4 trials) | $1 | 1 | $1 | 2 | $1 | 1 |
|  | $2 | 2 | $2 | 0 | $2 | 2 |
|  | $3 | 1 | $3 | 2 | $3 | 1 |
| **NOISE (9 trials)** | | | | | | |
|  | Payoffs: | Count | Payoffs: | Count | Payoffs: | Count |
| Correct | *Rand*($1:$9) | 7 | *Rand*($1:$9) | 7 | *Rand*($1:$9) | 7 |
| Incorrect | *Rand*($1:$9) | 2 | *Rand*($1:$9) | 2 | *Rand*($1:$9) | 2 |

**Participant’s Performance and Payoffs (Choice Manager):**

In the Choice condition, participants’ payoffs came from the same underlying pool of payoffs used in the No-Choice condition (for correct/incorrect trials). Noise trials were also included in each block at the same rate, further equating the two conditions.

If participants never learn any of the sequence, they may **choose completely at random**: they have a 25% chance of being correct on any given trial, because there are only 4 options (i.e., decks) to choose from among on a particular trial. Overall earnings will be approximately $3.53:

Overall E(V) Choice *Random Guessing (25% Accuracy):*

*Correct Incorrect*

$3.53 = [.23($7.63) + .02($5)] + [.69($2) + .06($5)]

*signal noise signal noise*

If the participant **learns about a third of the sequence (approximately 34% accuracy)**, then their average earnings will be approximately $4.01:

Overall E(V) Choice *(34% Accuracy):*

*Correct Incorrect*

$4.01 = [.31($7.63) + .03($5)] + [.62($2) + .05($5)]

*signal noise signal noise*

If the **participant learns two thirds of the sequence (approximately 67% accuracy)**, then their average earnings will be approximately $5.75:

Overall E(V) Choice *(67% Accuracy):*

*Correct Incorrect*

$5.75 = [.62($7.63) + .05($5)] + [.31($2) + .03($5)]

*signal noise signal noise*

### **S1.2.3 Manager Statements of Autonomy-Support vs. Coercion**

This is the list of autonomy-supportive, versus coercive, statements made by the managers (at random) during the choice versus no-choice trials.

**Choice Manager:**

"I value your input. What deck do you suggest?"

"I'm just here for your support. Don't mind me."

"I appreciate your perspective on this. Please choose for yourself."

"Feel free to handle this decision yourself."

"You can select any deck you like."

**No-Choice Manager:**

"I'm not interested in your opinion. Choose Deck #."

"Your input is not wanted. I say choose Deck #."

"When I'm in charge, decisions must be made through me. Choose Deck #."

"Decisions must be approved by me, your boss. Select Deck #."

## S1.3 Psychological Measures

**Self-Efficacy**

Please indicate how much you agree, or disagree, with the following statement about Part 1 of the Card Task, which you just completed:

1. I was able to do a good job when I made decisions myself.
2. I feel like I am good at this part of the card task.

*11-point scale (1 strongly disagree, 6 neutral, 11 strongly agree)*

**Difficulty**

1. How difficult was the card task that you just completed?

*11-point scale (1 Very easy, 6 neutral, 11 Very difficult)*

**PERCEPTIONS OF THE MANAGERS:**

**Self-Determination**

When I worked with the [Day/Night] Manager…

1. I had personal freedom.
2. I felt free to live life according to my desires.
3. My deck selections were determined by my own actions.
4. I determined what would happen during each deck choice.

*11-point scale (1 strongly disagree, 6 neutral, 11 strongly agree)*

**Procedural Fairness**

1. I felt like the manager used a just process to manage the decision situation.
2. I felt like the manager used a fair process to manage the decision situation.

**Interpersonal Fairness**

1. I felt like I was treated politely.
2. I felt like I was treated respectfully.

**General Fairness**

1. I felt like I was treated justly.
2. I felt like I was treated fairly.

**Security**

1. The card task felt well-structured and predictable.
2. I felt safe from uncertainties.
3. I felt a sense of security.

**Efficacy of the Manager:**

1. I was confident the manager and I would get good payoffs.
2. I was confident the manager and I would earn the best monetary outcomes possible.
3. I was confident the manager and I would make accurate decisions.
4. I was confident the manager and I would choose the right deck.

# S2.0 SUPPLEMENTAL ANALYSES

In this section, we provide supplemental analyses: noteworthy order effects and detailed table of descriptive statistics for outcome satisfaction.

## S2.1 Order Effects

As reported below, a few noteworthy effects emerged for order: participants felt more efficacious in Order 2, when the easier task followed the harder task; they also preferred the Choice Manager a bit more in Order 2 (most likely due to the enhanced feeling of self-efficacy they felt in Order 2).

**Efficacy:**

Overall, there was order effect between-participants, *F*(1,75)=10.75, *р*=.002, η*_p_*^2^=.13. On average, participants felt more efficacious in Order 2 (Order 1: *M*=5.64, *95%*CI[5.18,6.10]; Order 2: *M*=6.73, *95%*CI[6.25,7.25]). There was not an Order × Task Difficulty interaction, *F*(1,75)=0.93, *р*=.338, η*_p_*^2^=.01. We believe the noted, overall (average), effect of order is due to a anchoring and perceptual contrast effects. Specifically, in Order 2, Part 1 begins with the harder task, so participants have no prior reference with which to gauge or compare their efficacy. This lack of comparison results in a somewhat heightened (inflated) perception of efficacy initially. During Part 2, we believe a perceptual contrast effect occurs such that when participants experience the substantially easier task, this experience results in an artifically heightened feeling of efficacy (i.e., a bias in calibration: cf. Burger, 1989).

**Fig S1. Self-efficacy as a function of order and task difficulty.**


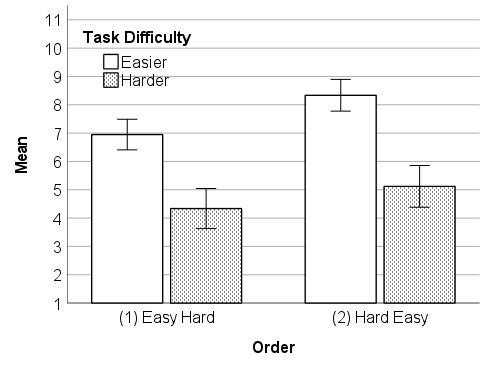


Error Bars: 95%CIs

**Percentage Preference for Choice:**

Overall, there was order effect between-participants, *F*(1,75)=6.95, *р*=.010, η*_p_*^2^=.09. On average, a greater number of participants preferred the Choice Manager in Order 2 (Order 1: *M*=55.00%, *95%*CI[45.59%,64.41%]; Order 2: *M*=72.97%, *95%*CI[63.19%,82.76%]). However, this effect is qualified by a significant Order × Task Difficulty interaction, *F*(1,75)=4.15, *р*=.045, η*_p_*^2^=.05. Specifically, as shown in Figure S.2, compared to Order 1, participants in Order 2 exhibited a heightened preference for Choice particularly in the harder task (Order 1 easier: *M*=82.50%, *95%*CI[70.94%,94.06%] vs. Order 2 easier: *M*=86.49%, *95%*CI[74.47%,98.50%]; Order 1 harder: *M*=27.50%, *95%*CI[12.55%, 42.45%] vs. Order 2 harder: *M*=59.46%, *95%*CI[43.92%, 75.00%]). This effect is likely due to the inflated sense of efficacy individuals felt in Order 2 (see Figure S.1).

**Fig S2. Preference for choice as a function of order and task difficulty.**


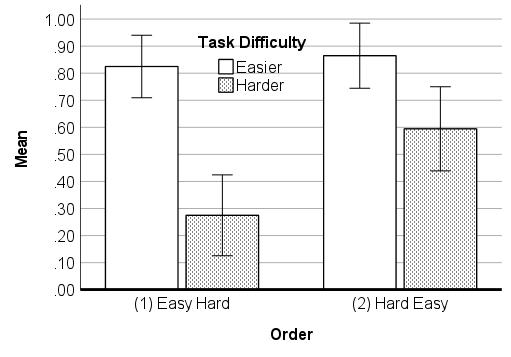


Error Bars: 95%CIs

## S2.2 Outcome Satisfaction

In this section, we report descriptive statistics (Table S5) and planned comparisons for outcome satisfaction.

**Table S5. Descriptive statistics for outcome satisfaction (mean, standard error, 95% confidence intervals) for each payoff, as a function of choice.**

|  | **Choice** | | **No-Choice** | |
| --- | --- | --- | --- | --- |
| **Outcome** | *Mean (SE)* | *95% CI* | *Mean (SD)* | *95% CI* |
| $1 | 1.62 (0.18) | 1.34, 2.04 | 1.64 (0.17) | 1.30, 1.98 |
| $2 | 2.21 (0.16) | 1.89, 2.53 | 2.07 (0.15) | 1.78, 2.37 |
| $3 | 2.87 (0.17) | 2.53, 3.20 | 2.71 (0.15) | 2.42, 3.00 |
| $4 | 3.77 (0.15) | 3.49, 4.06 | 3.71 (0.16) | 3.39−4.03 |
| $5 | 5.69 (0.13) | 5.44, 5.94 | 5.50 (0.12) | 5.27, 5.73 |
| $6 | 6.70 (0.14) | 6.43, 6.97 | 6.45 (0.14) | 6.17, 6.71 |
| $7 | 7.96 (0.14) | 7.69, 8.24 | 7.58 (0.15) | 7.28, 7.88 |
| $8 | 9.04 (0.15) | 8.73, 9.34 | 8.79 (0.15) | 8.49, 9.10 |
| $9 | 10.16 (0.15) | 9.87, 10.45 | 9.88 (0.18) | 9.53−10.22 |

**Planned Comparisons:**

Comparing Choice vs. No-Choice at each outcome:

- $1, *t*(76)=0.61, *p*=.544, *Cohen’s d*=0.07
- $2, *t*(76)=1.49, *p*=.139, *Cohen’s d*=0.17
- $3, *t*(76)=1.61, *p*=.111, *Cohen’s d*=0.18
- $4, *t*(76)=0.57, *p*=.572, *Cohen’s d*=0.07
- $5, *t*(76)=2.55, *p*=.013, *Cohen’s d*=0.29
- $6, *t*(76)=2.78, *p*=.007, *Cohen’s d*=0.32
- $7, *t*(76)=3.97, *p*<.001, *Cohen’s d*=0.45
- $8, *t*(76)=2.25, *p*=.027, *Cohen’s d*=0.26
- $9, *t*(76)=2.55, *p*=.013, *Cohen’s d*=0.29
